# The Development of a Multiple-Item Annoyance Scale (MIAS) for Transportation Noise Annoyance

**DOI:** 10.3390/ijerph15050971

**Published:** 2018-05-12

**Authors:** Dirk Schreckenberg, Christin Belke, Jan Spilski

**Affiliations:** 1 ZEUS GmbH, Centre for Applied Psychology, Environmental and Social Research, Sennbrink 46, 58093 Hagen, Germany; belke@zeusgmbh.de; 2 Center for Cognitive Science, University of Kaiserslautern, Erwin-Schrodiger-Straße, Building 57, 67663 Kaiserslautern, Germany; jan.spilski@sowi.uni-kl.de

**Keywords:** noise annoyance, ICBEN, MIAS, psychometric, reliability, validity, confirmative factor analysis, NORAH

## Abstract

In 2001, Team#6 of the International Commission on Biological Effects of Noise (ICBEN) recommended the use of two single international standardised questions and response scales. This recommendation has been widely accepted in the scientific community. Nevertheless, annoyance can be regarded as a multidimensional construct comprising the three elements: (1) experience of an often repeated noise-related disturbance and the behavioural response to cope with it, (2) an emotional/attitudinal response to the sound and its disturbing impact, and (3) the perceived control or coping capacity with regard to the noise situation. The psychometric properties of items reflecting these three elements have been explored for aircraft noise annoyance. Analyses were conducted using data of the NORAH-Study (Noise-Related Annoyance, Cognition, and Health), and a multi-item noise annoyance scale (MIAS) has been developed and tested post hoc by using a stepwise process (exploratory and confirmatory factor analyses). Preliminary results were presented to the 12th ICBEN Congress in 2017. In this study, the validation of MIAS is done for aircraft noise and extended to railway and road traffic noise. The results largely confirm the concept of MIAS as a second-order construct of annoyance for all of the investigated transportation noise sources; however, improvements can be made, in particular with regard to items addressing the perceived coping capacity.

## 1. Introduction

In 1988, Fidell et al. [1] drew a memorable picture when they said that noise-induced “annoyance is a chameleon-like concept that eludes succinct definition” (p. 13). Guski et al. [2] provide definitions of noise annoyance as used in different field and laboratory studies, and show a wide range of understanding regarding the concept of noise annoyance. This range includes noise annoyance defined as an emotion, an attitude, or knowledge, as well as a result of disturbance or rational decision. For example, Guski et al. [2] refer to Fidell [3], who describes annoyance as a rational decision in which respondents “balance one thing against another, they weigh different circumstances of their situation” [2] (p. 515). Further, Fidell et al. [1] (p. 13) specify that annoyance “is neither a sensation nor a physical quantity, but rather an attitude, a covert mental process with emotional and cognitive components. It is usually thought of as a generalized adverse attitude toward noise exposure […]”.

Some authors [4,5] found a significant correlation between the fear of aircraft accidents and noise annoyance, therefore allowing annoyance to be classified as an emotion. Leonard and Borsky [6] (p. 691) reported that “annoyance significantly increases or decreases with noise exposure only to the extent that fear [of aircraft operation] and health concern also increase or decrease”.

Additionally, in some studies and articles, the concept of noise annoyance is defined as a multidimensional construct. Consequently, Guski et al. [2] conclude that noise annoyance is a “psychological concept which describes a relation between an acoustic situation and a person who is forced by noise to do things he/she does not want to do, who cognitively and emotionally evaluates this situation and feels partly helpless” (p. 525), therefore defining noise annoyance as a “multifaceted concept”. In a recently published World Health Organisation (WHO) review on environmental noise annoyance by Guski et al. [7], it is defined as a “complex response” that consists of “an often repeated disturbance due to noise […] and is often combined with behavioural responses in order to minimise disturbances” (p. 1539). Also, noise annoyance is both an attitudinal and a cognitive response. In a slightly different approach, Kroesen and Schreckenberg [8] identified noise annoyance as one dimension of a general noise reaction. Further dimensions are activity disturbance, as well as feelings of fear and anxiety.

Over the years, different scales and questions have been used to assess noise annoyance, therefore making a comparison between different studies and/or cultures nearly impossible. In 2001, the Team#6 of the International Commission on Biological Effects of Noise (ICBEN) recommended two “multiple-purpose items” [9] for measuring noise annoyance (in the English version comprising “bother, disturb, annoy…”) in community noise surveys. One was a five-point verbal scale question, and the other was a 0–10 point numeric scale question developed for different languages. Two years later, these two annoyance scales recommended by ICBEN were adopted as the technical specification ISO/TS 15666:2003 of the International Organization for Standardization [10].

These items were foremost intended to enable the comparison of study results nationally and internationally through providing “a high-quality, reliable measure of a general reaction to a noise experienced in a residential environment” [9] (p. 643). Both items have been widely accepted in the scientific community and delivered their purposes. However, Job et al. [11] (p. 940) argue that questions “that ask only about annoyance fail to measure many possible and important reactions to noise. For example, people may react to noise with anxiety, distraction, exhaustion, anger, frustration, disappointment and fear”. Also, the aforementioned understanding of noise annoyance as a multidimensional construct is giving rise to the assumption that a single item does not represent noise annoyance accurately.

We argue that a multi-item noise annoyance scale implies the different facets of noise annoyance as described above. Further, the multi-item scale leads to a better differentiation between the different parts of noise annoyance that might be differently associated with acoustical and non-acoustical factors [1]. In his model of noise annoyance, Stallen [12] conceptualises annoyance as a psychological stress response to noise (stressor) with the primary appraisal of the degree of sound-induced perceived disturbances, and the secondary appraisal of perceived resources to cope with the noise (perceived control). Following this model, one would expect the disturbance part of annoyance to be more correlated with acoustical indicators of noise exposure and the non-acoustical factors, in particular those referring to the perception of control of the noise situation (e.g., noise sensitivity, perceived predictability of the noise, trust in authorities, see Stallen [12] for a detailed discussion) to be higher correlated with the annoyance aspect of perceived control or the capacity to cope with noise. In line with this, it is assumed that changes in annoyance over time [13] and/or the impact of stepwise changes in noise exposure on annoyance [14] might be better explained by analysing changes of the different aspects included in the multidimensional annoyance construct that is operationalised by a multi-item annoyance scale. Still, the multi-item scale is not meant to replace the single annoyance items recommended by the ICBEN [9,10], but is thought to be a comprehensive supplement.

Following Guski and colleagues [2,7], we believe that the multidimensional construct of noise annoyance comprises (1) the experience of repeatedly occurring noise-related disturbances and the behavioural response to cope with it, (2) an emotional/attitudinal response to the sound and its disturbing impact, (3) the perception of loss of control of the noise situation, or in other words, the perceived lack of capacity to cope with noise. To develop a multi-item annoyance scale that is meant to assess these different dimensions of noise annoyance, we conducted post-hoc analyses using data of work package 1 (WP1) of the NORAH-Study (Noise-Related Annoyance, Cognition, and Health) [15]. Within WP1 of this research initiative, the impact of transportation noise on noise annoyance and health-related quality of life (HQoL) has been studied. NORAH-WP1 includes a panel study at Frankfurt Airport (FRA) on the impact of aircraft noise on annoyance, reported sleep disturbances, and HQoL with measurements before (2011) and repeatedly after (2012, 2013) the opening of a new (fourth) runway (runway Northwest), and the implementation of a ban on night flights from 11 pm to 5 am (both in October 2011). Furthermore, WP1 entails cross-sectional studies on the impact of noise by railway and road traffic on the aforementioned outcomes in the vicinity of the airports Berlin-Brandenburg (BER) in 2012, Cologne/Bonn (CGN), and Stuttgart (STR), the latter two in 2013, as well as in the vicinity of Frankfurt Airport.

## 2. Materials and Methods

### 2.1. Study Design and Samples

For aircraft noise, surveys were carried out at the German airports Frankfurt (FRA), Berlin-Brandenburg (BER), Cologne/Bonn (CGN), and Stuttgart (STR). The definition of the study area around Frankfurt Airport differed between sub-studies, depending on the source of the transportation noise of interest. For aircraft noise, the study area was curtailed by the “envelope” of the 40-dB contours of the continuous aircraft sound levels for daytime (*L_p_*_Aeq,06-22h_) and night-time (*L_p_*_Aeq,22-06h_). Within this area, for the aircraft noise panel study (“FRA-air”), adult residents were randomly sampled from population registries in 2011 with (1) aircraft sound levels (2.5-dB classes of the maximum of *L_p_*_Aeq,06-22h_ and *L_p_*_Aeq,22-06h_ calculated for 2007, and (2) the change in aircraft sound exposure, i.e., the difference between address-related estimated *L_p_*_Aeq,24h_ as predicted for 2020 and *L_p_*_Aeq,24h_ of 2007, categorised in three groups (increase in *L_p_*_Aeq,24h_ >2 dB, decrease in *L_p_*_Aeq,24h_ >2 dB, change within the range of ±2 dB) as strata. Telephone numbers that were available from telephone registration were assigned to the sampled residents to enable telephone interviews as the main mode of the survey. The continuous sound levels used as stratum and to define the perimeter of the study region were calculated for the residential address of each participant, and refer to the air traffic of the six busiest months of the year 2007. Similarly, the sound levels predicted for 2020 refer to the six busiest months of 2020.

The cross-sectional study designs for the other airports—BER, CGN, and STR—follow the described design at Frankfurt Airport concerning the stratified random sampling with aircraft sound levels as stratum. The CGN and STR airports belong to the category of “low-rate-of-change” (LRC) airports, i.e., there is no indication of a step change in aircraft noise exposure three years before and after the survey [16]. The surveys at CGN and STR took place in 2013. The BER airport, similar the FRA airport, is a “high-rate-of-change” airport that was expecting changes at the time of the survey. That is, the regional airport, Berlin-Schoenefeld, was expected to be extended to the international BER (new runway and terminals). In the beginning of the NORAH study, the opening of BER was expected for 2012, but for several technical and organisational reasons, the new airport did not open during the whole period of the NORAH study (2011–2015). The ‘before measurement’ at the airport BER took place in 2012.

For road traffic and railway noise, two separate cross-sectional studies (“FRA-road” and “FRA-rail”, respectively) were carried out in the Rhine-Main region around Frankfurt Airport. The study design and sampling followed the concept of the aircraft noise surveys. Within the study area defined for the aircraft noise panel study (“FRA-air”), all of the addresses with the maximum of *L_p_*_Aeq,06-22h_ and *L_p_*_Aeq,22-06h_ for a road traffic sound exposure of 40 dB and higher were included in the pool for sampling for the sub-study “FRA-road”. Similar, all of the addresses with the maximum of *L_p_*_Aeq,06-22h_ and *L_p_*_Aeq,22-06*h*_ for railway sound exposure of at least 40 dB were included in the address pool for the sub-study ‘FRA-rail’. The sampling criteria of “FRA-road” and “FRA-rail”, respectively, included the criterion of “dominance of the noise source of interest”. This criterion guaranteed that the average sound pressure level *L_p_*_Aeq,24h_ of the respective noise source under study was at least 2.5 dB *L_p_*_Aeq,24h_ higher than the average sound pressure level *L_p_*_Aeq,24h_ of any of the other existing transportation noise sources. Within a stratified random sampling procedure, the samples were then drawn at random using source-specific 2.5-dB classes of the maximum of the address-related continuous sound levels for daytime (*L_p_*_Aeq,06-22h_) and for the night (*L_p_*_Aeq,22-06h_) as stratum. For “FRA-road”, the stratum refers to road traffic sound levels, for “FRA-rail”, it refers to railway sound levels, respectively. All of the sound levels for the sample stratification and exposure-response analyses were calculated on the basis of official sound computation regulations (see Section 2.3, below). Table 1 depicts the samples at the four airports.

The development and psychometric testing of the noise annoyance scale was first done for aircraft noise with data of the ‘FRA-air’ sample after the changes at the airport in the most recent measurement in 2013. In addition, the construct validity of the developed scale was tested with the data of the samples at the other airports, see also Schreckenberg et al. [17]. In this study, the scale was also adopted to annoyance due to road traffic and railway noise with data of the cross-sectional surveys ‘FRA-road’ and ‘FRA-rail’, respectively. All in all, the validity of the multiple-item scale for noise annoyance was tested for aircraft, road traffic, and railway noise, with a total sample of *N* = 20469.

### 2.2. Procedure

The participants of the ‘FRA-air’ panel study at Frankfurt Airport were sampled in the spring of 2011. The sampling of the participants at BER airport was done in the spring of 2012, and the sampling for CGN and STR were done in the summer of 2013. The respondents of the studies ‘FRA-road’ and ‘FRA-rail’ were sampled in 2012. All of the sampled residents received a cover letter to inform about the study and invite participation in telephone interviews or optional online surveys with the same questionnaire. In the sub-study ‘FRA-air’, the first measurement was done in the summer and autumn of 2011, and finished before the opening of the runway Northwest on 21 October 2011. Repeated measurements were carried out in the summer/autumn of 2012, and again in 2013. The measurement at BER took place from May to August 2012, those around CGN and STR took place between August and December 2013. The surveys ‘FRA-rail’ and ‘FRA-road’ were carried out in the autumn of 2012. The sampling and data management was supervised and certified by each responsible agency for data protection.

### 2.3. Noise Exposure

The exposure to transportation sound levels for each participant’s residential address (continuous and mean maximum sound levels of aircraft, railway, and road traffic) was calculated for a 12-month period between October and September for each survey wave for daytime, evening, night-time, and for 24 h. For the assessment of aircraft sound levels, the German calculation method AzB 2008 was used [18]. The average sound levels of railway and road traffic for a 12-month period were determined based on the methods for calculation used for European Union (EU) noise mapping [19,20]. For the analyses in this study, the *L*_den_ and the *L_p_*_Aeq_ for 24 h, at daytime (6 am–10 pm), and night-time (10 pm–6 am) as indicators of aircraft, road traffic, and railway sound exposure were used. See Möhler et al. [21] for more information about the address-related estimation of transportation sound levels in the NORAH study. For different noise metrics, Table 2 shows the wide range of exposure to aircraft, railway, and road traffic noise in the samples of this study.

### 2.4. Questionnaire

In all of the surveys at every airport, the questionnaires included the assessment of disturbances and annoyance to aircraft noise and other transportation noise sources (railway, road traffic), the capacity to cope with noise, HQoL, potential co-determinants of annoyance (e.g., noise sensitivity, attitudes towards the source and towards authorities, expectations), questions concerning residential conditions (e.g., sound insulation, window type and position), and demographics. In the analyses described in this paper, source-specific noise annoyance was assessed with the ICBEN five-point annoyance scale [9] according to ISO/TS 15666 [10]. Similarly, for the assessment of noise disturbances referring to activities of communication, relaxing/concentration, and sleep, the verbal markers of the ICBEN five-point scale were used. The (lack of) perceived coping capacity was assessed with six items on a five-point rating scale (see Table 3 and Supplementary Materials Table S1).

The HQoL was ascertained by means of the SF-8, which is a short form of the standardised SF-36 [22]. The SF-36 is a measure of generic functional health status and well-being that was used to assess eight domains of HQoL [23,24,25,26]. HQoL, again, can be “viewed as self-report relating to psychological, social, physical, and everyday life components of well-being and function as perceived by the person” ([23], p. 191). We used the eight items of the shorter form SF-8 [27] in order to measure eight health domains: general health (GH), physical functioning (PF), physical role (RP), bodily pain (BP), vitality (VT), social functioning (SF), emotional role (RE), and mental health (MH) for the period of four weeks prior to the interview. The item scores were transformed to T values with *M* = 50 and *SD* = 10, and summed up to two sum scores of HQoL, the mental component score (MCS), and the physical component score (PCS), according to the QualityMetric’s scoring algorithms [22].

The variable ‘expectations concerning the impact of air traffic on the regional development and the residential life’ was assessed by a mean score of the following items on a five-point scale (agree (1) not–(5) very): (1) the airport has improved regional development; (2) air traffic leads to a fall in the value of residences and properties; (3) air traffic brings new jobs to the region; (4) air traffic spoils residents’ outdoor stays in the garden, on the terrace, or on the balcony. Cronbach’s alpha for the four-item expectation scale is α = 0.74. As the variable name and the content of the items would indicate, the items only refer to aircraft noise, and were not used in the sub-studies ‘FRA-road’ and ‘FRA-air’.

For the assessment of ‘trust in authorities’, the participants were asked to rate the extent of authorities’ endeavours to minimise residents’ noise annoyance. The authorities that were judged in this way were source-specific. That is, for aircraft noise (samples ‘FRA-air’, ‘BER-air’, ‘CGN-air’, and ‘STR-air’) these were the aircraft manufactures, airlines, the airport operator, the regional aircraft noise commission, German Air Traffic Control, municipalities, the regional dialogue forum ‘Forum Airport & Region’ (only ‘FRA-air’), the respective Federal State Government, the aircraft noise commissioner, and the Federal Aviation Office. For road traffic noise (‘FRA-road’), these were car manufacturers, regional public transport companies, municipalities, the Hessian Authority for Road and Transportation (Hessen Mobil), the Federal Highway Research Institute, Federal State Government, and car drivers. For railway noise (‘FRA-rail’), the judged authorities were rolling stock manufacturers, Deutsche Bahn AG, (German railway, Berlin, Germany), regional public transport companies, municipalities, and the Federal State Government. The authorities’ endeavours were assessed on a five-point response scale from (1) ‘not’ (5) ‘very’. The source-specific authorities were judged rather homogeneously, allowing the definition of summarised (source-specific) scores of ‘trust in authorities’. The judgements for some of the authorities were excluded from the scoring of ‘trust in authorities’ because of a number of missing responses ≥10%. For the other items, the mean scores of source-specific ‘trust in authorities’ were calculated (Cronbach’s alpha for trust in five aircraft-related authorities: α = 0.84 in ‘FRA-air’, 2013; for five road traffic-related authorities: α = 0.81 in ‘FRA-road’; and for three railway-related authorities, α = 0.80 in ‘FRA-rail’. The items included in the scoring are listed in Table S2 of the Supplementary Materials.

Other questions that were included in the questionnaire referred to residential conditions (e.g., house ownership, window type and position, duration of living in the residential area) and residential satisfaction, self-reported diagnoses of several physical and mental health diseases, medication use, risk factors (e.g., smoking, drinking alcohol, physical activity), and socio-demographics. In addition, in the panel sample ‘FRA-air’, further questions specifically refer to the (expected, perceived) changes since the opening of the new runway in October 2011.

### 2.5. Statistical Analyses

The multiple-item noise annoyance scale (MIAS) was first developed and tested exemplarily for aircraft noise (MIAS-air). The tests were done with regard to the psychometric quality (construct validity, reliability) of the scale in a stepwise process using data of the last measurement in 2013 of the FRA panel sample. Exploratory factor analysis (EFA), second-order confirmatory factor analysis (CFA), and the calculation of Cronbach’s alpha were conducted. The analyses were done in six main steps. Preliminary results referring in particular to MIAS-air are described also by Schreckenberg et al. [17].

1. By content, we collected a list of 21 items (see Table 3) that were regarded as potentially reflecting the three elements of noise annoyance as defined by Guski and colleagues [2,7]. Other items included in the questionnaire on residential conditions/satisfaction, reported health diseases, changes in aircraft noise exposure at Frankfurt Airport, and demographics (see Section 2.4) were not considered as being part of the multidimensional annoyance concept.
2. We used EFA (principle axis factoring: PAF) with oblique rotation conducted in SPSS to examine the factor structure. As the criterion to determine the number of factors, we employed Kaiser’s criteria (eigenvalue > 1 rule). The initial PAF was conducted on the list of 21 items. The global suitability of the respondent data for PAF was very good (*KMO* >0.90, Bartlett’s test of sphericity, *p* <0.01). However, successive tests indicated that several items were unsuitable for factor analysis due to low communalities (*h*^2^ <0.30; items: I-10, I-12, I-14, I-15), or low factor loadings (<0.50; items: I-5, I-6, I-7, I-8, I-11, I-13, see Table 3). In sum, we excluded 10 items (48%). The item selection aimed at maximising parsimony and achieving a number of items below 10 for the assessment of the components of noise annoyance as identified by Guski and colleagues [2,7]. Therefore, the final PAF was conducted with 10 items, plus the ICBEN item (five-point scale). Following, the selected set of items was submitted to a final EFA in order to examine the factor structure. As this set of items still includes altogether more than 10 items, the final selection was done by content (see Section 3.1), resulting in a set of six items plus the ICBEN annoyance item.
3. Confirmatory factor analysis (CFA) was performed to investigate whether the factor structure was of psychometric adequacy. Therefore, a second-order CFA was conducted in Mplus with one and three factors, with and without correlated error terms. The CFA were carried out with robust maximum likelihood estimation (*MLR*) and the imputation of missing values with the *FIML* algorithm (full information maximum likelihood estimation). In the second order CFA, a general aircraft noise annoyance score including the sub-dimensions ‘aircraft noise-related disturbances’, annoyance (the ICBEN annoyance item), and ‘lack of coping capacity’ was modeled in accordance with the definition by Guski and colleagues [2,7] in order to test the construct validity and reliability of the annoyance scale. Two CFA versions were compared: (1) with one annoyance factor including all of the items (model A), and a hierarchical structure with three factors (F1 ‘disturbances’; F2 ‘lack of coping capacity’; and the item ‘annoyance’ on the ICBEN five-point scale) (model B). In addition, a third CFA model with the three factors of model B presented separately was calculated for control. With this latter model, it was tested whether a hierarchical model structure with MIAS as a second-order factor (model B) is superior to a model where the factors F1, F2, and the ICBEN item are assumed to correlate without forming a common underlying second order factor (model C). That is, model C would indicate that F1, F2, and the ICBEN annoyance item are distinct although interrelated psychological concepts. Actually, the results show that the model fit indices are better for model C than for model B, which might be because the available items were chosen post hoc to form the MIAS factor, and that a priori tailored items would improve the multiple-item annoyance scale. For this paper, the analyses focus on testing whether MIAS appears to be an improvement compared with a single annoyance item, and whether MIAS would be better reflected by a one-factor structure (model A) or a hierarchical second-order structure (model B). For results referring to model C, see Chapter 2 in the Supplementary Materials. Beside the test statistics for the CFA models, model fit was evaluated using (1) the comparative fit index (*CFI*) for which values above 0.90 indicate an acceptable fit, and values of 0.95 and higher indicate a very good fit; (2) the root mean square error approximation (*RMSEA*) for which values of 0.05 and less indicate a very close fit, and values of 0.08 and less still an acceptable close fit; (3) the standardised root mean square residual (*SRMR*) for which values below 0.10 are recommended [28,29,30]. The reliability of the latent constructs (factors F1 and F2) was assessed with the composite reliability (*CR*) coefficient. The internal consistency of each set of items that together measure the constructs F1 and F2, respectively, was assessed with Cronbach’s alpha. The convergent validity was ascertained with the average variance extracted (*AVE*). *CR* values ≥ 0.6 [31] and a Cronbach’s alpha value of ≥0.7 indicate an acceptable reliability of the factors. *AVE* values should be >0.5 for acceptable convergent validity [32].
4. For aircraft noise, the MIAS’s construct validity was analysed by comparing the results gained from the ‘FRA-air’ sample data with the CFA results of the data of the samples at the BER, CGN, and STR airports. In addition, the construct validity of MIAS was analysed for road traffic noise annoyance (MIAS-road) and railway noise annoyance (MIAS-rail) by means of a CFA of the data of the sub-studies ‘FRA-road’ and ‘FRA-rail’, respectively.
5. The new developed MIAS-air, MIAS-road, and MIAS-rail scores and their components were correlated with acoustical and non-acoustical factors, and variables of HQoL that are known to be related to noise annoyance. This was done in order to evaluate the criterion validity of the annoyance assessment.
6. In order to test whether MIAS can be regarded as an improvement of noise annoyance measurement in models of the relationship between noise and health outcomes, regression analyses of the SF8 scores MCS and PCS on MIAS (regression 1) and on the ICBEN noise annoyance item (regression 2) were carried out.

## 3. Results

### 3.1. Selection of Items for the Aircraft Noise Annoyance Scale

Twenty-one items extracted from the questionnaire were submitted to an initial EFA (principle axis factoring, PAF, with oblique rotation). Table 3 shows a list of these items.

Similar items were also included in the questionnaires of the sub-studies that focused on road traffic and railway noise, respectively (‘FRA-road’, ‘FRA-rail’)—see Table S1 in the Supplementary Materials. The item list was pre-selected, and the items were preliminarily grouped by content in the categories mentioned by Guski and colleagues [2,7]. As criteria for the item selection results of the initial EFA analysis referring to measures of sample adequacy (MSA), communalities and factor loading were used in addition to selection by content. For example, during the process of item selection, items of reported sleep disturbances were excluded because of low factor loadings (<0.50). However, in this study, an exclusion of these items by content would also be justified: at the different airports studied in NORAH-WP1, there are different regulations/restrictions of flight operations at night-time. Thus, an annoyance score including reported sleep disturbances would mean a different psychological concept at different airports. Probably, this is reflected in the low factor loadings.

A reduced set of 10 items together with the ICBEN aircraft noise annoyance item was again submitted to a final EFA. The Kaiser–Meyer–Olkin coefficient (*KMO* = 0.916) and the Bartlett test (χ² = 26311.53, *df* = 55, *p* < 0.001) indicate the adequacy of the included items. The EFA extracted one factor (eigenvalue >1) that explains 55.21% of variance. Forcing EFA to extract two factors (in addition to the ICBEN annoyance item) revealed an explained variance of 69.39%. The two identified factors can be labeled according to the components of noise annoyance mentioned in Guski et al. [7] as ‘aircraft noise-related disturbances (F1)’ and ‘perceived lack of coping capacity (F2)’. Table 4 shows the factor loadings of the included items (without the ICBEN annoyance item).

As the aim was to develop a parsimonious measure of noise annoyance with less than 10 items, we selected three items of factor F1 (disturbances) and three items of factor F2 (lack of coping capacity) to form the MIAS together with the ICBEN noise annoyance item. This final selection was done by content. From the list of 10 items (see Table 4), item F1.4 was excluded, as it reflects active indoor communication, which is already addressed by item F1.1. From the list of items aimed at reflecting a perceived lack of coping capacity/loss of control, items F2.4 to F2.6 were removed. This was decided because, by content, F2.1 to F2.3 seem to reflect more the notion of perceived (loss) of control over the noise situation and the perception of to what extent there is the capacity to actively cope with the noise problem, whereas items F2.4 to F2.6 seem to refer more to implemented passive, cognitive coping strategies. Items F2.4 to F2.6 have a somewhat ambiguous connotation. It is not clear whether individuals switch off (F2.4), do not hear the noise anymore (F2.5), or have accepted the presence of noise (F2.6) because this is the way they control the person—noise environment relation, or because they have resigned. Statistically, the six items of coping capacity couldn’t be distinguished in different factors; thus, to finalise a selection of 10 items, the items with potentially more ambiguous meanings were dropped from the set of MIAS items.

According to the EFA results, a hierarchical factor structure of the components of the multiple aircraft annoyance scale is suggested. This structure was tested by means of confirmatory factor analysis (CFA) for the final selected seven items: three items for each factor F1 and F2 and the ICBEN annoyance item.

### 3.2. CFA for Aircraft Noise Annoyance Assessed at Frankfurt Airport

Two CFA models were performed to study the psychometric adequacy of the factor structure of MIAS. The first one (model A) includes one factor comprising all seven items. The second one (model B) consists of a hierarchical structure of the factors F1, F2, the ICBEN annoyance item, and the second-order factor MIAS. In model B, residual co-variance was estimated between the items ‘disturbance talk/phone’ and ‘disturbance TV, radio’, as both items reflect the sub-concept of communication disturbances. Similar, a residual co-variance was modelled between the items ‘protect myself against noise’ and ‘close the window’, as they are formulated positively in terms of still having the capacity to cope with the noise, and they are both introduced in the models in an inversely (negatively) re-coded version.

For the sample ‘FRA-air’, Table 5 shows the indices of the CFA model fit. Further results of the CFA analysis performed with data of sample 'FRA-air' are shown in the Supplementary Materials, Tables S3–S6, and Figures S1–S3. The indices in Table 5 indicate a sufficient model fit in particular for model B. This suggests a hierarchical structure of the multidimensional annoyance concept with disturbances (F1) and lack of coping capacity (F2) forming together with the ICBEN annoyance item the higher order construct ‘annoyance’.

**Figure 1:**
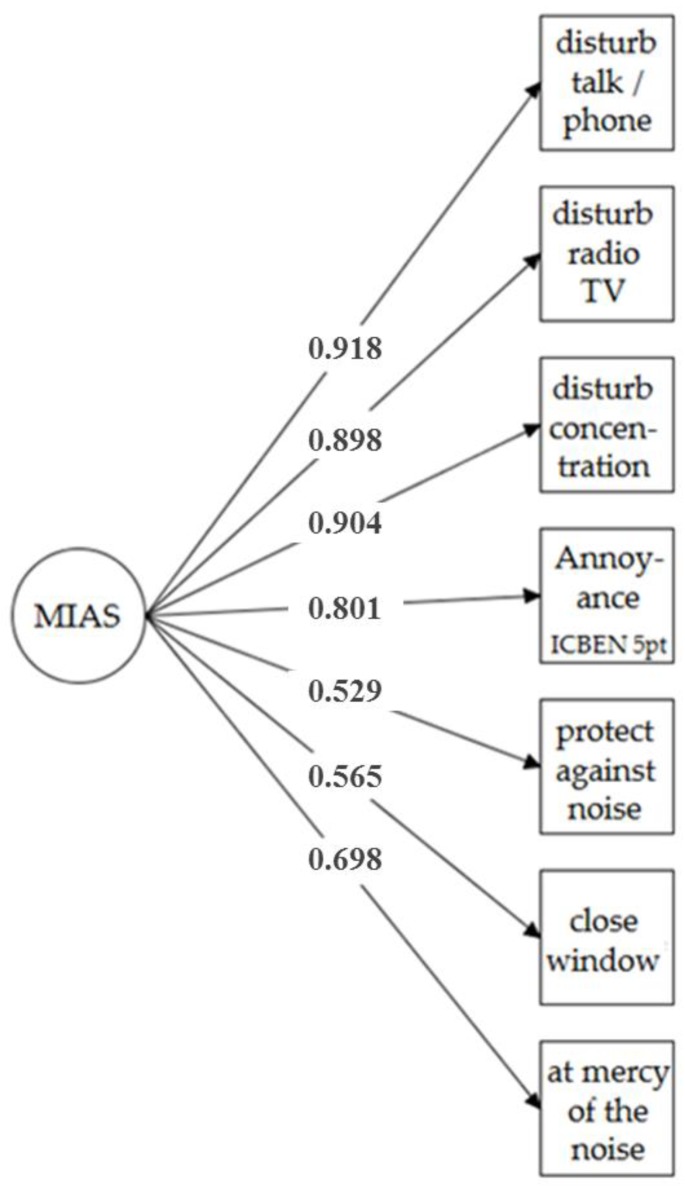
Confirmatory factor analysis (model A) of a one-factor multiple item score for aircraft noise annoyance (MIAS-air) as measured in the sample at Frankfurt Airport in 2013 (*n* = 3508).

**Figure 2:**
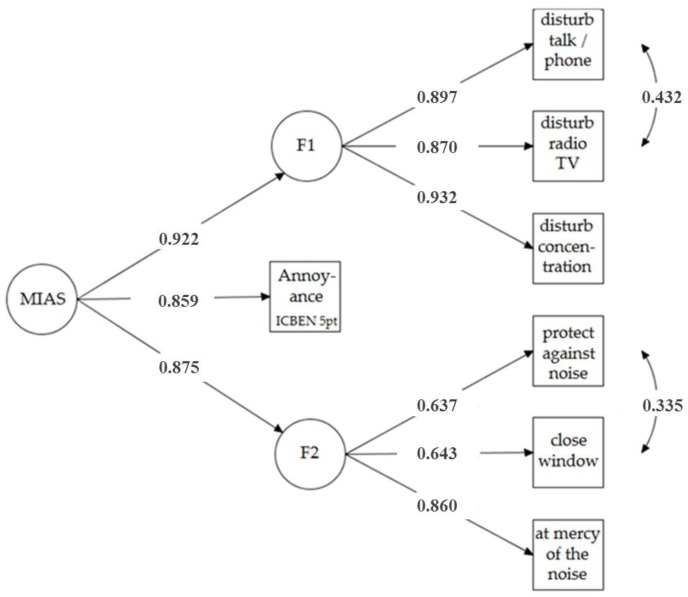
Confirmatory factor analysis (model B) of higher order multiple item score for aircraft noise annoyance (MIAS-air) as measured in the sample at Frankfurt Airport in 2013 (*n* = 3508).

The reliability scores *CR* and Cronbach’s alpha (α) suggest a good reliability of the MIAS constructs (*CR* = 0.76 to 0.92; α = 0.79 to 0.94), the *AVE* suggests a good convergent validity (*AVE* = 0.52 to 0.80 in model B). This justifies the hierarchical structure, i.e., it indicates that both factors seem to belong to the same second-order factor. Cronbach’s alpha for the total MIAS (model A), including items of the factors F1 and F2 and the ICBEN annoyance item, is α = 0.91. In particular, model B provides a better fit to the data than a one-factor model does (model A) without a hierarchical structure (Satorra-Bentler Δχ² (3) = 986.88, *p* < 0.001). Figure 1 and Figure 2 depict the CFA models A and B.

### 3.3. Comparison of CFA Results for Aircraft, Railway and Road Traffic Noise Annoyance

Results of CFA analyses for the samples at the FRA, BER, CGN, and STR airports for aircraft, railway, and road traffic noise annoyance are given in the Supplementary Materials, Tables S7–S15, and Figures S4–S9. The model fit values for MIAS as assessed at the other airports confirm the results of the aircraft noise annoyance assessment at Frankfurt Airport (Supplement Materials, Tables S7, S10, S14): for the samples at all of the airports, the indices reflect a better fit for model B than for model A (Supplementary Materials, Table S7). In line with this, the factor loading of the items on factors F1 and F2 and the loadings of F1, F2 and the ICBEN annoyance item on the second-order factor MIAS are in a similar range in the samples at the different airports (Table 6). This suggests that the hierarchical structure of MIAS has a satisfying construct validity for aircraft noise annoyance beyond the sample at Frankfurt Airport.

The model fit values for MIAS as assessed for railway noise (Supplementary Materials, Table S10) and road traffic (Supplementary Materials, Table S14) are, altogether, in a similar range than those for aircraft noise (Supplementary Materials, Table S7). That is, the CFA results on MIAS for railway and road traffic noise broadly confirm the satisfying construct validity of the hierarchical structure of MIAS.

However, the factor loading of the items on factors F1 and F2, and the loadings of F1, F2, and the ICBEN annoyance item on the second-order factor MIAS are not always in the same range for railway and road traffic noise compared with those for aircraft noise (Table 6). In particular, the factor loadings of the F2 items F2.1 (‘protect against noise’) and F2.2 (‘close windows’) on F2 are lower for railway and road traffic noise than for aircraft noise. Accordingly, in the MIAS models for railway and road traffic noise annoyance (model B), the factor F2 loads lower on MIAS than in the model for aircraft noise annoyance (Table 6). In addition, the reliabilities (*CR*, α) and the convergent validity (*AVE*) of the factor F2 ‘Lack of coping capacity’ as well as the correlations of F2 with the other factors (Table 7) are somewhat lower in the MIAS models for road traffic and railway noise than in the model for aircraft noise.

### 3.4. Correlations of MIAS with Acoustical, Non-Acoustical Factors, and Outcomes of Health-Related Quality of Life

As measurements of criterion validity, correlations were calculated between MIAS and its components and variables known from previous studies to be associated with annoyance. Earlier studies have shown that noise annoyance is associated with long-term average sound levels and non-acoustical factors such as noise sensitivity and attitudes towards the source or towards authorities (e.g., Guski [33]) as well as with sleep disturbances and with health-related quality of life (HQoL) (e.g., Shepherd et al. [34]). If MIAS is a valid measurement of noise annoyance, a similar correlation pattern between noise annoyance and the external acoustical and non-acoustical criteria is expected for MIAS and the ICBEN five-point annoyance item. Therefore, comparisons of the correlation structure of noise annoyance as measured with the ICBEN five-point annoyance item, factors F1 and F2, and the MIAS score reflecting these three components were made. The correlation analyses were done for aircraft noise annoyance (with data of the sub-study ‘FRA-air’), for road traffic noise annoyance (with study data of ‘FRA-road’), and for railway noise (with study data of ‘FRA-rail’).

For the correlation analyses MIAS, F1 and F2 were estimated as latent variables according to the CFA model B. Results are shown in Table 8 for aircraft noise annoyance, in Table 9 for railway noise annoyance, and in Table 10 for road traffic noise annoyance.

For aircraft noise, both MIAS and the ICBEN annoyance item are correlated with indicators of aircraft sound exposure in a similar range, although the correlation coefficients of MIAS with the exposure indicators are slightly higher than those of the ICBEN annoyance item (Table 8). The highest correlation coefficients are observed for MIAS and the factor F1, ‘disturbances’. The correlations of F2, ‘lack of coping capacity’, with the exposure indicators are lower compared with the other annoyance components.

Table 8 also shows that MIAS-air and the ICBEN annoyance item largely correlate with non-acoustical factors with a quite similar effect size. However, MIAS-air correlates slightly higher with the non-acoustical factors than the ICBEN annoyance item. As expected, factor F1, ‘disturbances’ (at daytime), correlates somewhat higher with sleep disturbances than factor F2. Furthermore, F1 correlates higher with the judgment of air traffic as useful, comfortable for users, and environmentally harmful, and with PCS than factor F2. Factor F2, ‘lack of coping capacity’, correlates somewhat higher with the judgment of air traffic as dangerous, expectations regarding the air traffic, trust in authorities, MCS, and noise sensitivity.

A quite similar picture can be found for railway noise annoyance (Table 9), although in total, the annoyance variables correlate slightly lower with the acoustical and non-acoustical variables than the variables referring to aircraft noise.

For road traffic noise (Table 10), the correlations of the MIAS-road annoyance scores, ICBEN annoyance item, F1, and F2 with most of the acoustical and non-acoustical variables are considerably lower than those observed for aircraft and railway noise. There is mixed evidence regarding whether either MIAS-road or the ICBEN annoyance item correlate higher with noise metrics, sleep disturbances, non-acoustical factors, or HQoL variables. In particular, factor F1, ‘disturbances’, shows lower correlations with the acoustical and non-acoustical factors compared with F1 and the equivalent variables for aircraft and railway noise. As for railway and aircraft noise, factor F2, ‘lack of coping capacity’ correlates somewhat higher with ‘trust in authorities’, noise sensitivity, and MCS than factor F1.

All in all, the correlation coefficients suggest a satisfying criterion validity of MIAS and the components F1 and F2. In this study, the structure of associations with acoustical and non-acoustical factors, sleep disturbances, and HQoL are altogether quite similar to that of the single ICBEN annoyance item, and to what is known from the literature. This is true for aircraft and railway noise, and with a somewhat lower effect size for road traffic noise.

### 3.5. Regressions of HQOL Variables MCS and PCS on Aircraft Noise Annoyance and Exposure (L_den_)

In research on noise effects, scientists endeavour to get more insight into the relationship between noise annoyance and further mental and physical health outcomes [35,36]. For this, an improvement in the measurement of noise annoyance for the prediction of health is of interest. Whether MIAS can be regarded as such an improvement is tested by means of regression analyses of the SF8 scores MCS and PCS on MIAS-air and the day–evening–night level *L*_den_ (regression 1a, 2a), and on the ICBEN aircraft noise annoyance item and *L*_den_ (regression 1b, 2b) with data of the sample ‘FRA-air’.

As Table 11 shows, in all of the regression models, annoyance contributes considerable more to the explanation of the SF8 scores than the *L*_den_. In three of the four regression analyses, the contribution of the aircraft noise exposure as expressed by *L*_den_ is not significant. For both measures of annoyance—MIAS and the ICBEN item—more of the variance of the mental component score (MCS) is explained compared with the explained variance of the physical component score (PCS). Furthermore, the contribution (beta) of aircraft noise annoyance is higher for the prediction of MCS than for the prediction of PCS. However, more variance of the SF8 scores MCS and PCS is explained in regression models where MIAS-air is included, compared with regression models with the single ICBEN annoyance item. In addition, the regression with MIAS-air results in a significant increment in *R*^2^ (for PCS Δ*R*^2^ = 0.012, for MCS Δ*R*^2^ = 0.049).

## 4. Discussion

### 4.1. The Factorial Structure of MIAS

In this study, a multi-item scale for noise annoyance (MIAS) preliminary that was developed for aircraft noise-related annoyance [17] has been extended to transportation noise, i.e., aircraft, railway, and road traffic noise, and tested with regard to its reliability and validity. With the development of MIAS, we follow the definition of noise annoyance as a multidimensional psychological construct [2].

For the analyses, we used data on community responses to transportation noise that was collected in the years 2012 and 2013 at the four German airports Frankfurt, Berlin-Brandenburg, Cologne/Bonn, and Stuttgart within the frame of the NORAH research initiative (Noise-Related Annoyance, Cognition, and Health). We intended to develop and test a reliable and valid parsimony scale, including a number of less than 10 items, to allow its use in field studies. According to Guski et al. [2,7] noise annoyance includes at least three elements: (1) the often repeated experience of disturbances, which are often combined with behavioural responses to reduce the disturbances; (2) an emotional/attitudinal response to the sound and its disturbing impact; and (3) a cognitive response that implies the perceived lack of capacity to cope with noise. We selected six items, plus the ICBEN five-point annoyance item, and analysed their factorial structure.

Through confirmative factor analyses (CFA), we tested two models: (A) a one-factor structure with MIAS as a factor, including items of disturbances, annoyance and the lack of capacity to cope with noise; and (B) a hierarchical structure with the second-order multiple-item noise annoyance scale (MIAS) consisting of two factors F1 (‘noise-related disturbances’), F2 (‘perceived lack of coping capacity’), and the single ICBEN five-point annoyance item.

It was found that for the assessment of transportation noise annoyance, MIAS as a second-order construct (model B) is a reliable scale of satisfying construct and criterion validity across different sources of transportation noise (aircraft, railway, road traffic) and, with regard to aircraft noise, across surveys at different German airports. This is in line with the statement of Guski et al. [2,7] that annoyance includes the three elements of perceived noise disturbances, an emotional/attitudinal response, and the perceived loss of control (or lack of coping capacity). The results are also confirmed by a study of Kroesen et al. [37], who presented a structural equation analysis of aircraft noise annoyance on the basis of Stallen’s framework for environmental noise annoyance [12]. These authors regard noise annoyance as a psychological stress response to noise that is influenced by perceived noise disturbance and perceived control and coping capacity. However, Kroesen et al. [37] and Stallen [12] regard the perceived disturbance and control/coping capacity as concepts separate from, though (reciprocally) related to noise annoyance.

MIAS, which has been modelled as one factor (model A), already has a good internal consistency according to Cronbach’s alpha (0.84 < α < 0.91 for aircraft noise annoyance, α = 0.82 for road traffic noise annoyance, and α = 0.84 for railway noise, respectively). Nevertheless, the results of different CFA models suggest that the model fit improves considerably when MIAS is modelled as a second-order construct (model B). That is, model B is superior to model A. This is true for all of the studied transportation noise sources and—for aircraft noise—all of the surveys at different airports.

### 4.2. Factor F2: ‘Lack of Coping Capacity’

The factor loadings of the CFA models indicate that factor F2, ‘Perceived lack of coping capacity’, and its items are the ‘weak elements’ in the factorial structure of MIAS. This is particularly true for the first two items, F2.1 ‘Protect against noise (re-coded)’ and F2.2 ‘Close windows (re-coded)’. In the questionnaires, these items are formulated positively (see also Table S1 in the Supplementary Materials): I-16 (F2.1), ‘I know that I can protect myself quite well against noise’, and I-17 (F2.2), ‘If it is too loud outside, I simply close the windows, and then I am no longer disturbed’. All of the other items, the third F2 item, F2.3, and the disturbance items of F1 and the ICBEN annoyance item, are formulated in a negative sense. For the analyses in this study, the F2 items F2.1 and F2.2 were inversely re-coded. It is likely a methodological artefact of the re-coding that the two items do not fit well to the factorial structure of MIAS. Probably, these items do not reflect the notion of lack of perceived control as the third F2 does (I-18 (F2.3): ‘Sometimes, I really feel at the mercy of the noise.’), and thus do not represent the third element of the concept of annoyance as defined by Guski et al. [2]. There are different styles of coping with stress to be differentiated (e.g., problem-oriented versus emotion-oriented coping styles [38]). This holds true for environmental stressors, and noise in particular [39]. The F2 factors refer to the perception of coping capacity. Following the content of the F2 items, it can be assumed that the first two items address more a problem-oriented, proactive coping style, whereas the third one addresses a more emotion-oriented, reactive coping style. That is, the three F2 items address different aspects of coping, and thus might be differently related to the concept of annoyance. In line with this, uncontrollability and unpredictability are key aspects of the definition of a stimulus (sound) as a stressor (noise). Noise responses, in particular disturbances and annoyance, are known to be associated with loss of control and the (un)predictability of noise situations [2,12,40,41]. The loss of control, if not its intensified form, helplessness, is more expressed in item F2.3 (I-18) than in the other two F2 items. This, again, might explain why the item F2.3 has a higher factor loading than the items F2.1 and F2.2 in the factorial structure of the annoyance scale. A further reason that the factor F2 has a lower factor loading than F1 in all of the CFAs might be that the F2 items are not formulated source-specifically. That is, the ICBEN annoyance and the disturbance items refer to either aircraft, railway, or road traffic noise, respectively, whereas the F2 items refer to ‘general opinions about noise’ (see Table S1 in the Supplementary Materials). This is due to one of the main limitations of this study: that MIAS has been developed post hoc with already collected data.

In line with this, the reference of the F2 items to noise in general across noise sources might also explain why the factor loading of F2 on MIAS is lower for railway and road traffic noise than for aircraft noise. It might be that residents living in the vicinity of an international airport have aircraft noise in mind when responding to these general noise-related questions on perceived coping capacity, even when they are also asked for annoyance and disturbance due to railway and road traffic noise, respectively.

### 4.3. The Criterion Validity of MIAS

MIAS fits with the concept of annoyance as a stress response to noise according to the stress concept of Lazarus, as adopted by Stallen [12]. Factor F1, showing—at least for aircraft and railway noise—the highest correlation with exposure indicators among the components of MIAS, seems to reflect the primary appraisal of the stressor ‘noise’ and factor F2 (lack of coping capacity), the secondary appraisal of available coping resources. The correlation matrix concerning the non-acoustical factors suggests that the association between annoyance and the non-acoustical factors, in particularly the expectations concerning the impact of air traffic, trust in authorities, and noise sensitivity, refer more to the secondary appraisal, i.e., the capacity to cope with noise, which is in line with the noise annoyance model presented by Stallen [12]. This is not consistently true for the judgments of attributes of the transportation noise source. The reason might be a methodological one. That is, in the NORAH study [15], the attribute items were expected to reflect the underlying construct ‘Attitudes towards the noise source’. However, psychometric analysis revealed that for each noise source, the attributes were judged too heterogeneously to form a reliable consistent score of attitude. Therefore, this heterogeneity might also be expressed in different relationships to the different components of annoyance.

MIAS turns out to be an improved measure of noise annoyance compared with the single ICBEN annoyance item in analyses on the relationship between noise and HQoL. This was shown in regression models for the SF-8 scores for physical (PCS) and mental well-being (MCS).

Furthermore, PCS is quite similarly correlated with the factors F1 and F2 for aircraft and road traffic noise annoyance, and is somewhat higher for F2 than F1 for railway noise annoyance. MCS is more strongly associated with the factor F2 in the correlation analyses for all of the sources of transportation noise. This might indicate different mechanisms of the mediation effect of noise annoyance on self-reported health (see also Schreckenberg et al. [42]). Repeatedly experienced sound-related disturbances, combined with the perception of being less able or unable to cope with it, might lead to physical arousals and hinder recovery from noise-induced (physiological) stress, and thus impair physical health on a long-term basis. Mental well-being might be reduced because of the perception that one cannot get rid of the noise, whereas mental health might be less negatively affected even when an individual experiences higher noise disturbances, as long as the person still perceives to have enough resources and options etc. to cope with it. This might have implications for different noise control strategies, either to improve mental or physical well-being. However, to study this in more detail is out of the scope of this study.

For all of the studied transportation noise sources, noise sensitivity is also more highly correlated with factor F2, ‘lack of coping capacity’ than with Factor F1, ‘noise-related disturbances’. Noise sensitivity is regarded as a stable trait; it is an attitude, or an internal state that, while independent from noise exposure, increases the susceptibility of an individual to noise in general and hence mediates or moderates reactions to noise [43,44,45]. Among others, noise sensitivity is known to affect noise annoyance [43,44] and sleep disturbances [46,47], and is associated with mental health complaints [48,49].

The fact that noise sensitivity correlates with a perceived lack of coping capacity, which can also be interpreted as the noise-specific perception of loss of control [50], is in line with Hatfield [41] and Stansfeld [48], who reported that noise-sensitive people perceive noise as being more out of their control. That noise sensitivity correlates less with factor F1, ‘disturbances’, than with factor F2 indicates that self-reported noise sensitivity is less related to the disturbance due to the stressor ‘transportation noise’ as to the insight that an individual is less able to overcome this disturbance, and thus get the situation of being disturbed under control. However, at this stage, this is just a speculation on the basis of correlations, and needs further investigation. Nevertheless, in this regard, a result of the above-mentioned SEM analysis of aircraft noise annoyance by Kroesen et al. [37] is interesting. The authors assumed aircraft noise annoyance to be influenced by perceived disturbance and perceived control and coping capacity. They also included noise sensitivity in their initial model, and assumed that perceived disturbance is positively influenced by noise sensitivity. Kroesen et al. [37] found no statistically significant influence of noise sensitivity on perceived disturbance, and excluded this variable from the final model. This result seems to support the assumption derived from the results of our study that noise sensitivity is less associated with disturbances, and is instead more related to perceived control and coping capacity.

### 4.4. MIAS Versus Single-Item Annoyance Assessment

All in all, the CFA results encourage the idea of the assessment of noise source-specific disturbances and perceived coping capacity in addition to a single ICBEN noise annoyance item in order to grasp the multiple dimensionality of the concept of annoyance. In practice, this means that scores for F1 and F2 should be calculated before summarising these scores together with the ICBEN annoyance item to MIAS. From a statistically point of view, a higher-order factor of annoyance consisting of the two factors F1 and F2 and the ICBEN annoyance item would be a reliable and valid as well as a parsimonious construct. At least, it is recommended to consider F1 and F2 in addition to the single annoyance item, even when not summarising them to a second-order score.

To continue the internationally standardised assessment of noise annoyance, the inclusion of the single annoyance item(s) suggested by ICBEN [9] is still recommended. The ICBEN annoyance item used in this study to assess noise annoyance itself fits well into the factorial structure of MIAS. Moreover, the single five-point ICBEN noise annoyance item was found to be a noise annoyance assessment of good criterion validity. That is, correlations of aircraft, railway, and road traffic noise annoyance as measured with the single ICBEN five-point annoyance item with acoustical and non-acoustical factors, variables of sleep disturbances, and HQoL are of expected size and quite similar to those of MIAS.

However, one of the advantages of the noise annoyance assessment as a multiple-item second order construct is that it helps to get a deeper understanding of the interrelations between different noise effects, and thus might be more effective in the assessment of the impact of noise-related interventions (changes in exposure in terms of improvement due to noise abatement or worsening, e.g., due to expansion of infrastructure). Also, with six plus one items, MIAS turns out to be quite efficient and suitable for the use in socio-acoustical field studies.

Another advantage of MIAS compared with a single annoyance item is that the association between noise annoyance and non-acoustical factors is often interpreted in terms of the non-acoustical factors engendering a response bias in annoyance judgments [1], sometimes even intentionally, in order to foster activities of responsible authorities to reduce the noise. With a set of multiple items to assess annoyance, this response bias is expected to be reduced, and in addition, different causes of different components of annoyance are more explicit. This conclusion was already drawn when presenting the first results of the development of MIAS for aircraft noise annoyance [17]. In this study, we could show that, in principle, this seems to hold true for the assessment of transportation noise annoyance in general.

### 4.5. Limitations

The study has several limitations. First, the development and validation of MIAS has been done post hoc, although it is theoretically driven. That is, in the NORAH study, when developing the questionnaires and the items, it was not aimed to assess annoyance with multiple items to operationalise its multidimensional character. Among others, this means that the aspect of affective reaction to the noise as mentioned by Guski et al. [2,7] could not be operationalised by items directly referring to emotional reactions, as no explicit emotion-related item concerning aircraft, railway, and road traffic noise, respectively, was assessed. A related item was used assessing the perception of the noise source as dangerous (I-13, Table 3). However, this item addresses an emotional/attitudinal response to the source, not to the noise of this source. Furthermore, in the initial EFA, the factor loading of this item was too low (<0.05) and, therefore, the item was excluded from further analyses. Instead, for each transportation noise source, the ICBEN annoyance five-point item was used as its own ‘proxy’ for an affective reaction to noise. In addition, as mentioned, the items of lack of coping capacity were not formulated specifically with reference to the transportation noise source of interest, but to noise (at home) in general. This might have led to lower factor loadings compared with the disturbance and annoyance items. In the future, we encourage the development of noise-source specific items that operationalise the lack of coping capacity as well as source-specific items reflecting the emotional responses to the noise.

Second, the chosen items of activity disturbances (items of factor F1) refer to activities at daytime (communication, concentration). In this study, sleep disturbance was not included in the MIAS because of low factor loadings and because otherwise, the different nocturnal flight operations at the investigated airports (part of the airports have night flight restrictions) would lead to different psychological constructs of MIAS and factor F1 at the different airports. However, this means that an important noise-related disturbance associated with the annoyance judgement [37,51,52] is excluded from the annoyance assessment. On the other hand, some authors model self-reported sleep disturbance as being modified or mediated by annoyance [53,54,55], probably indicating the respondents’ difficulties with judging (in surveys taken place at daytime) disturbances taken place at night-time. That is, although there is no doubt that noise-related sleep disturbance is associated with annoyance, the causal direction of the link is not fully clear.

Third, in this study, the numerical 11-point scale recommended by ICBEN Team#6 [9,10] was not used. Therefore, it is unclear how this item would fit into the factorial structure of MIAS.

Fourthly, MIAS was first developed for aircraft noise annoyance, and then adopted to railway and road traffic noise of residents living in the vicinity of an international airport (here: Frankfurt Airport). Within the frame of the NORAH project in a study on the impact of combined noise on total annoyance, Wothge et al. [56] showed that in the Rhine-Main Region around Frankfurt Airport, aircraft noise was the perceived dominant (most annoying) noise source in this region, and that railway and road traffic noise hardly contributed to the total annoyance judgments. Therefore, it might be that the salience of air traffic (noise) in the region around Frankfurt Airport has affected the understanding of the non-source-specific items of factor F2 in the samples addressing road traffic and railway noise, respectively (FRA-road, FRA-rail). To some extent, this might have weakened the reliability of the MIAS factor F2, ‘lack of coping capacity’, for road traffic and railway noise annoyance. Thus, for future research, we suggest (a) the use of source-specific items (see the first limitation mentioned above) and (b) investigating other study areas with less salience of a further noise source (here: aircraft) in order to confirm and improve the construct validity of MIAS for railway and road traffic noise.

## 5. Conclusions

In this study, data on annoyances due to transportation noise collected in WP1 of the NORAH research program (Noise-Related Annoyance, Cognition, and Health) was analysed in order to develop a multiple-item annoyance scale (MIAS) and test it with regard to its reliability and validity. For this, exploratory and confirmative factor analyses (EFA, CFA) have been carried out to identify the factorial structure of MIAS for aircraft noise annoyance within a sample at Frankfurt Airport, and test the psychometric quality of the scale for aircraft noise annoyance with data collected in samples at three other German airports (Berlin-Brandenburg, Cologne/Bonn, and Stuttgart), and for railway and road traffic noise with data of separate cross-sectional studies collected in the Rhine-Main-Region around Frankfurt Airport. By means of correlation analyses with acoustical, non-acoustical factors, variables of sleep disturbances, and HQoL, which are known to be associated with annoyance, the criterion validity of MIAS and its components were investigated. Whether MIAS is an improved measure of noise annoyance for analyses of the relationship between noise and health was studied by means of regression analyses for the SF8 scores PCS and MCS.

As a main result of the assessment of source-specific annoyance due to transportation noise, the reliable multi-item annoyance scale MIAS could be identified as a second-order construct of satisfying construct and criterion validity across the noise sources aircraft, railway, and road traffic. With six plus one items, the MIAS is quite efficient and applicable for use in field studies. For aircraft noise annoyance, the reliability and validity of the MIAS and its components could be confirmed in different samples at the different German airports that were included in the NORAH study. Although the further use of the single annoyance item(s) as suggested by ICBEN [9] is still recommended in order to continue the internationally standardised assessment of noise annoyance, and although our results show that the five-point ICBEN annoyance item turned out to be a noise annoyance assessment of good validity, the use of a multiple-item annoyance scale still is of advantage. It helps to get more insight into the complex structure of annoyance and its relations to acoustical and non-acoustical factors. Furthermore, it increases the explained variance of self-reported physical and mental well-being. At least, it is suggested to assess noise-related disturbances and perceived control and coping capacity in addition to single annoyance items even when not summarised to a second-order score.

As the MIAS was developed post hoc with the best items *available* in the NORAH-WP1 study, the composition of the score is of satisfying quality, but it is also far from perfect. For future research, it is suggested to develop and include more noise source-specific items of (loss of) perceived control and (lack of) coping capacity, as well as source-specific emotional noise responses in addition to the single ICBEN annoyance items and items on noise disturbances in order to develop a multiple-item annoyance scale of better psychometric quality.

## Figures and Tables

**Table 1:** Samples of Noise-Related Annoyance, Cognition, and Health (NORAH) surveys at the Frankfurt (FRA), Berlin-Brandenburg (BER), Cologne/Bonn (CGN), and Stuttgart (STR) airports.

Airport—Noise Source of Interest	Year of Measurement/Sample Size			Gender	Age (In Last Year of Measurement)			
	2011	2012	2013	% Female	*Min*	*Max*	*Med*	*M (SD)*
‘FRA-air’	9244	4867	3508	53.5	20	98	54	54.6 (14.6)
‘BER-air’		5548		52.1	18	100	60	57.9 (15.5)
‘CGN-air’			2955	51.5	18	95	60	58.7 (16.2)
‘STR-air’			1979	50.5	18	97	60	58.5 (15.7)
‘FRA-road’		3172		51.7	18	100	59	57.4 (16.0)
‘FRA-rail’		3307		51.3	19	92	59	57.4 (15.7)
Total	20469							

Note: *Min* = minimum; *Max* = maximum; *Med* = median; *M* = mean; *SD* = standard deviation.

**Table 2:** Exposure to aircraft noise in the ‘FRA-air’, ‘BER-air’, ‘CGN-air’, and ‘STR-air’ samples, to road traffic noise in the ‘FRA-road’ sample, and to railway noise in the ‘FRA-rail’ sample.

Noise Metric		‘FRA-Air’	‘BER-Air’	‘CGN-Air’	‘STR-Air’	‘FRA-Road’	‘FRA-Rail’
		*n* = 3508	*n* = 5548	*n* = 2955	*n* = 1979	*n* = 3172	*n* = 3307
		Source of Noise Exposure					
		Aircraft	Aircraft	Aircraft	Aircraft	Road Traffic	Railway
*L_p_*_Aeq,06-22h_	*Min*	35.9	35.0	35.0	35.0	36.5	35.0
	*Max*	71.7	60.7	74.4	62.4	83.3	81.3
	*M*	48.6	43.9	46.4	45.2	58.9	57.8
	*SD*	6.3	6.7	7.2	7.7	9.6	8.7
*L_p_*_Aeq,22-06h_	*Min*	35.0	35.0	35.0	35.0	35.0	35.0
	*Max*	64.2	54.8	65.7	53.8	73.4	82.6
	*M*	41.6	39.8	46.3	38.8	50.9	58.8
	*SD*	5.9	5.1	7.2	4.6	9.5	9.0
*L_p_*_Aeq,24h_	*Min*	35.0	35.0	35.0	35.0	35.0	35.0
	*Max*	70.3	59.3	72.6	61.0	81.7	81.8
	*M*	47.2	42.9	46.5	43.9	57.4	58.3
	*SD*	6.3	6.4	7.1	7.5	9.6	8.7
*L*_den_	*Min*	38.1	35.0	35.0	35.0	37.8	35.0
	*Max*	73.8	63.1	74.2	64.2	84.0	88.7
	*M*	50.5	46.3	52.8	46.3	60.6	65.0
	*SD*	6.5	7.4	7.2	8.1	9.6	8.8

Note: *Min* = minimum; *Max* = maximum; *M* = mean, *SD* = standard deviation.

**Table 3:** Initial list of 21 items for the assessment of aircraft noise annoyance.

Aircraft Noise-Related Disturbances	Affective Evaluation, Attitudes	Perception of Loss of Control, Lack of Coping Capacity
In the last 12 months aircraft noise has disturbed … I-1 during communication, when using the phone at home. I-2 when listening to the radio and watching TV. I-3 when reading and concentrating. I-4 when having visitors at home. I-5 when staying and/or recovering outdoors. I-6 when falling asleep. I-7 during the night. I-8 when awakening. (1) not at all, (2) slightly, (3) moderately, (4) very, (5) extremely.	I-9 ICBEN five-point aircraft noise annoyance. Expectations concerning impact of air traffic on residential quality of life: Response scale: agree (1) not, (2) a little bit, (3) moderately, (4) rather, (5) very. I-10 Air traffic leads to a fall in the value of residences and properties. I-11 Air traffic spoils residents’ outdoor stays in the garden, on the terrace, or on the balcony. Attributes of air traffic:Response scale: agree (1) not, (2) a little bit, (3) moderately, (4) rather, (5) very. Air traffic is … I-12 useful. I-13 dangerous for me. I-14 comfortable for users. I-15 environmentally harmful.	Perceived capability to cope with noise: Response scale: agree (1) not, (2) a little bit, (3) moderately, (4) rather, (5) very I-16 I know that I can protect myself quite well against noise. I-17 If it is too loud outside, I simply close the windows, and then I am no longer disturbed. I-18 Sometimes, I really feel at the mercy of the noise. I-19 If it is very loud, I just mentally switch off. I-20 I do not hear the noise anymore. I-21 I have accepted the fact that the noise is here.

**Table 4:** Results of exploratory factor analysis (EFA) (principle axis factoring, or PAF) with forced extraction of two factors.

Item		Factor	
		F1—(Aircraft Noise-Related Disturbances)	F2—(Perceived Lack of Coping Capacity)
F1.1	In the last 12 months, aircraft noise has disturbed during communication, when using the phone at home	0.982	
F1.2	In the last 12 months, aircraft noise has disturbed when listening to the radio and watching TV	0.956	
F1.3	In the last 12 months, aircraft noise has disturbed when reading and concentrating	0.875	
F1.4	In the last 12 months, aircraft noise has disturbed when having visitors at home	0.927	
F2.1	I know that I can protect myself quite well against noise (recoded)		0.608
F2.2	If it is too loud outside, I simply close the windows, and then I am no longer disturbed (recoded)		0.589
F2.3	Sometimes, I really feel at the mercy of the noise	0.395	0.463
F2.4	If it is very loud, I just mentally switch off		0.747
F2.5	I do not hear the noise anymore		0.768
F2.6	I have accepted the fact that the noise is here		0.655

**Table 5:** Fit indices of confirmatory factor analyses (CFA), sample ‘FRA-air’ (*n* = 3508)—aircraft noise.

Model	Indicators	*χ*²	*df*	*p*	*CFI*	*RMSEA* (90% *CI*)	*SRMR*	*AIC*
A	MIAS-air, 1 factor	1582.786	14	<0.001	0.878	0.179 (0.171–0.186)	0.074	68,948.858
B	MIAS-air, three indicators (F1, annoyance, F2) and residual co-variances	100.413	11	<0.001	0.993	0.048 (0.040–0.057)	0.023	67,086.043

Note: *χ*²: Chi square test, *df*: degrees of freedom, *p* = probability of error, *CFI*: comparative fit index, *RMSEA*: root mean square error of approximation, 90% *CI* = 90% confidence interval, *SRMR*: standardised root mean square residual values, *AIC*: Akaike information criterion. MIAS: multi-item noise annoyance scale.

**Table 6:** Parameters of CFA for model B for MIAS conducted with data of the samples ‘FRA-air’, ‘BER-air’, ‘CGN-air’, ‘STR-air’, FRA-rail’, and ‘FRA-road’.

Estimates (Factor Loading)			‘FRA-Air’	‘BER-Air’	‘CGN-Air’	‘STR-Air’	‘FRA-Rail’	‘FRA-Road’
F1	<---→	disturb talk/phone	0.897	0.880	0.896	0.885	0.911	0.869
F1	→	disturb radio, TV	0.870	0.882	0.892	0.883	0.905	0.856
F1	→	disturb concentration	0.932	0.918	0.922	0.892	0.926	0.906
F2	→	protect against noise	0.637	0.507	0.567	0.532	0.558	0.491
F2	→	close windows	0.643	0.505	0.572	0.460	0.525	0.463
F2	→	at the mercy of the noise	0.860	0.793	0.847	0.800	0.914	0.924
MIAS	→	F1	0.922	0.899	0.876	0.964	0.943	0.986
MIAS	→	Annoyance, ICBEN 5-pt.	0.859	0.735	0.808	0.735	0.751	0.655
MIAS	→	F2	0.875	0.817	0.861	0.683	0.550	0.554

Note: *p* < 0.001 for all estimates. MIAS: multiple-item noise annoyance scale (for aircraft, railway, and road traffic, respectively). Annoyance, ICBEN 5-pt = 5-point annoyance scale as recommended by the International Commission on Biological Effects of Noise (ICBEN).

**Table 7:** Factors’ psychometric adequacy of MIAS model for aircraft (‘FRA-air’), railway (‘FRA-rail’), and road traffic noise (‘FRA-road’).

Source	Construct	*CR*	α	*AVE*	1	2	3
Air *N* = 3508	Disturbance	0.92	0.94	0.80	-		
	Lack of coping capacity	0.76	0.79	0.52	0.79 ***	-	
	Annoyance (single item)	-	-	-	0.80 ***	0.77 ***	-
Rail *N* = 3307	Disturbance	0.94	0.94	0.83	-		
	Lack of coping capacity	0.72	0.76	0.48	0.48 ***	-	
	Annoyance (single item)	-	-	-	0.73 ***	0.51 ***	-
Road *N* = 3172	Disturbance	0.91	0.92	0.76	-		
	Lack of coping capacity	0.68	0.73	0.45	0.50 ***	-	
	Annoyance (single item)	-	-	-	0.70 ***	0.51 ***	-

Note: *AVE* = average variance extracted; *CR* = composite reliabilities; α = Cronbach’s alpha; the remaining values indicate correlations between factors. *** *p* < 0.001.

**Table 8:** Correlation of aircraft noise annoyance (MIAS-air and its components) with indicators of non-acoustical aircraft sound exposure factors and SF8 scores of health-related quality of life (‘FRA-air’, *n* = 3506–3508).

Variables	MIAS-Air—Aircraft Noise Annoyance Score	Annoyance—Air (ICBEN 5-pt. Scale)	F1—Annoyance (Disturbances)—Air	F2—Annoyance (Lack of Coping Capacity)—Air
*L_p_Aeq,06-22h__*—air	0.520	0.466	0.512	0.355
*L_p_Aeq,22-06h__*—air	0.469	0.425	0.463	0.306
*L_p_Aeq,24h__*—air	0.519	0.466	0.510	0.353
*L*_den_—air	0.512	0.463	0.502	0.346
Disturbance falling asleep—air	0.786	0.669	0.733	0.682
Disturbance night sleep—air	0.601	0.502	0.729	0.501
Disturbance—awaken in morning—air	0.838	0.739	0.756	0.731
Air traffic is useful	−0.343	−0.294	−0.349	−0.318
Air traffic is dangerous for me	0.594	0.496	0.539	0.565
Air traffic is comfortable for users	−0.173	−0.149	−0.161	−0.142
Air traffic is environmentally harmful	0.359	0.315	0.342	0.327
Expectations conc. impact of air traffic	−0.744	−0.656	−0.624	−0.727
Trust in authorities—air	−0.491	−0.438	−0.397	−0.516
SF8 Physical Component Summary (PCS)	−0.192	−0.149	−0.187	−0.173
SF8 Mental Component Summary (MCS)	−0.319	−0.235	−0.291	−0.366
Noise sensitivity (single item)	0.347	0.258	0.285	0.348

Note: *L_p_*_Aeq_ = continuous sound level averaged over 12 months, *p* ≤ 0.001 for all of the correlation coefficients. MIAS = multiple-item annoyance scale, ICBEN 5-pt. scale = 5-point annoyance scale as recommended by the International Commission on Biological Effects of Noise (ICBEN), F1 = factor 1, F2 = factor 2.

**Table 9:** Correlation of railway noise annoyance (MIAS-rail and its components) with indicators of railway sound exposure, non-acoustical factors, and SF8 scores for health-related quality of life (‘FRA-rail’, *n* = 3307).

Variables	MIAS-Rail Railway Noise Annoyance Score	Annoyance—Rail (ICBEN 5-pt. Scale)	F1—Annoyance (Disturbances)—Rail	F2—Annoyance (Lack of Coping Capacity)—Rail
*L_p_Aeq,06-22h__*—rail	0.473	0.430	0.423	0.184
*L_p_Aeq,22-06h__*—rail	0.465	0.428	0.413	0.186
*L_p_Aeq,24h__*—rail	0.474	0.432	0.423	0.183
*L*_den_—rail	0.469	0.431	0.418	0.185
Disturbance falling asleep—rail	0.807	0.679	0.701	0.484
Disturbance night sleep—rail	0.757	0.637	0.654	0.460
Disturbance—awaken in morning—rail	0.775	0.646	0.680	0.477
Rail traffic is useful	−0.144	−0.121	−0.129	−0.098
Rail traffic is dangerous for me	0.407	0.330	0.366	0.287
Rail traffic is comfortable for users	−0.067	−0.040	−0.069	−0.072
Rail traffic is environmentally harmful	0.268	0.185	0.258	0.191
Trust in authorities—rail	−0.284	−0.267	−0.202	−0.228
SF8 Physical Component Summary (PCS)	−0.148	−0.131	−0.113	−0.188
SF8 Mental Component Summary (MCS)	−0.189	−0.139	−0.164	−0.256
Noise sensitivity (single item)	0.245	0.202	0.179	0.439

Note: *L_p_*_Aeq_ = continuous sound level averaged over 12 months, *p* ≤0.001 for all correlation coefficients. MIAS = multiple-item annoyance scale, ICBEN 5-pt. scale = 5-point annoyance scale as recommended by the International Commission on Biological Effects of Noise (ICBEN), F1 = factor 1, F2 = factor 2.

**Table 10:** Correlation of road traffic noise annoyance (MIAS-road and its components) with indicators of road traffic sound exposure non-acoustical factors and SF8 scores of health-related quality of life (‘FRA-road’, *n* = 3172).

Variables	MIAS-Road Road Traffic Noise Annoyance Score	Annoyance—Road Traffic (ICBEN 5-pt. Scale)	F1—Annoyance (Disturbances)—Road Traffic	F2—Annoyance (Lack of Coping Capacity)—Road Traffic
*L_p_*_Aeq,06-22h_—road	0.332	0.353	0.284	0.165
*L_p_*_Aeq,22-06h_—road	0.321	0.348	0.269	0.170
*L_p_*_Aeq,24h_—road	0.331	0.353	0.283	0.166
*L*_den_—road	0.328	0.352	0.279	0.168
Disturbance falling asleep—road	0.784	0.625	0.692	0.506
Disturbance night sleep—road	0.466	0.557	0.630	0.466
Disturbance—awaken in morning—road	0.773	0.629	0.680	0.490
Road traffic is useful	−0.102	−0.148	−0.109	−0.183
Road traffic is dangerous for me	0.307	0.250	0.278	0.249
Road traffic is comfortable for users	−0.055	−0.072	−0.082	−0.061
Road traffic is environmentally harmful	0.111	0.136	0.130	0.167
Trust in authorities—road	−0.128	−0.205	−0.179	−0.276
SF8 Physical Component Summary (PCS)	−0.141	−0.112	−0.127	−0.120
SF8 Mental Component Summary (MCS)	−0.238	−0.184	−0.217	−0.277
Noise sensitivity (single item)	0.271	0.214	0.215	0.431

Note: *L_p_*_Aeq_ = continuous sound level averaged over 12 months, *p* ≤0.001 for all of the correlation coefficients. MIAS = multiple-item annoyance scale, ICBEN 5-pt. scale = 5-point annoyance scale as recommended by the International Commission on Biological Effects of Noise (ICBEN), F1 = factor 1, F2 = factor 2.

**Table 11:** Results of regression: analysis for SF8 scores PCS and MCS (‘Fra-air’, *N* = 3.508).

Model 1	Criterion	Predictor	*R²*	*Beta*	*p*
1a	PCS	MIAS-air	0.034	−0.188	<0.001
		*L*_den_		0.006	0.732
1b	PCS	ICBEN aircraft annoyance item	0.022	−0.144	<0.001
		*L*_den_		−0.011	0.574
2a	MCS	MIAS-air	0.104	−0.343	<0.001
		*L*_den_		0.053	0.003
2b	MCS	ICBEN aircraft annoyance item	0.055	−0.240	<0.001
		*L*_den_		0.012	0.516

Note: PCS = physical component score, MCS = mental component socre, *L*_den_ = Day-Evening-Night level, MIAS = multiple-item annoyance scale, ICBEN = International Commission on Biological Effects of Noise.

## Supplementary Materials

The following are available online at http://www.mdpi.com/1660-4601/15/5/971/s1, Table S1: List of items for the assessment of aircraft noise annoyance and similar items referring to road traffic noise annoyance and railway noise annoyance, Table S2: Items of the non-acoustical variables correlated with noise annoyance (in addition to the list of items in Table S1), Table S3: Items MIAS scale (FRA-air, *N* = 3508), Table S4: Additional information—Final Factor loadings CFA (FRA-air, *N* = 3508), Table S5: Fit indices of CFA (FRA-air, *N =* 3508), Table S6: Factors’ psychometric adequacy (MIAS scale FRA-air, *N =* 3508), Table S7: Comparison of fit indices of CFA for the multiple-item aircraft noise annoyance scale (MIAS-air) conducted with data of the samples FRA, BER, CGN, and STR, Table S8: Items MIAS scale (railway)—FRA-rail, *N =* 3307, Table S9: Additional information—Final Factor loadings CFA (FRA-rail, *N =* 3307), Table S10: Fit indices of CFA (Railway, FRA-rail, *N =* 3307), Table S11: Factors’ psychometric adequacy (MIAS scale railway, FRA-rail, *N =* 3307), Table S12: Items MIAS scale (road, FRA-road, *N =* 3172), Table S13: Additional information—Final Factor loadings CFA (FRA-road, *N =* 3172), Table S14: Fit indices of CFA (road, FRA-road, *N =* 3172), Table S15: Factors’ psychometric adequacy (MIAS scale model road, FRA-road, *N =* 3172), Figure S1: CFA model A: Aircraft (FRA-air; *N =* 3508), Figure S2: CFA-model B: Aircraft (FRA-air; *N =* 3508), Figure S3: CFA-model C: Aircraft (FRA-air; *N =* 3508), Figure S4: CFA-model A: Railway (FRA-rail, *N =* 3307), Figure S5: CFA-model B: Railway (FRA-rail, *N =* 3307), Figure S6: CFA-model C: Railway (FRA-rail, *N =* 3307), Figure S7: CFA-model A: Road (FRA-road, *N =* 3172), Figure S8: CFA-model B: Road (FRA-road, *N =* 3172), Figure S9: CFA-model C: Road (FRA-road, *N =* 3172).
