# Supplementary material for: The Development of a Multiple-Item Annoyance Scale (MIAS) for Transportation Noise Annoyance
Source: Int J Environ Res Public Health. 2018 May 12;15(5):971. doi: 10.3390/ijerph15050971 (PMC5982010; doi:10.3390/ijerph15050971)
Supplement: Supplementary file 1 [file ijerph-15-00971-s001.pdf]

International Journal of
Environmental Research and Public Health

Supplement Material: The Development of A Multiple-item Annoyance Scale (MIAS) for Transportation Noise Annoyance

Dirk Schreckenberg, Christin Belke and Jan Spilski

1. NORAH Questionnaire items used in the analyses

Table S1. List of items for the assessment of aircraft noise annoyance and similar items referring to road traffic noise annoyance and railway noise annoyance.

<Source-specific> noise-related disturbances	Affective evaluation, attitudes	Perception of loss of control, lack of coping capacity
In the last 12 months, how much has <source-specific> noise disturbed you …<br><br>I-1. during communication, when using the phone at home<br>I-2. when listening to the radio and watching TV<br>I-3. when reading and concentrating<br>I-4. when having visitors at home<br>I-5. when staying and/or recovering outdoors<br>I-6. when falling asleep<br>I-7. during the night<br>I-8. when awakening<br><br>(1) not at all, (2) slightly, (3) moderately, (4) very, (5) extremely	I-9. a-c *ICBEN 5-point noise annoyance*<br><br>I-9a. Thinking about the last 12 months, when you are at home, how much does noise from road traffic disturb or annoy you? (1) not at all, (2) slightly, (3) moderately, (4) very, (5) extremely.<br>I-9b. And what about railway noise? Does it disturb or annoy you (1) not at all, (2) slightly, (3) moderately, (4) very, (5) extremely?<br>I-9c. And what about aircraft noise? Does it disturb or annoy you (1) not at all, (2) slightly, (3) moderately, (4) very, (5) extremely?<br><br>*Expectations concerning impact of air traffic on residential quality of life:*<br>Now, we come to general opinions about the airport <airport name> and its operations. Please tell me to what extent you agree to the following statements, i.e. whether you agree (1) not, (2) a little bit, (3) moderately, (4) rather, (5) very.<br><br>I-10. The air traffic leads to fall in value of residence and properties<br>I-11. The air traffic spoils residents'	*Perceived capability to cope with noise:*<br><br>Now, we come to general opinions about noise. Please tell me to what extent do you agree to the following statements. Please indicate whether you agree (1) not, (2) a little bit, (3) moderately, (4) rather, (5) very.<br><br>I-16. I know that I can protect myself quite well against noise.<br>I-17. If it is too loud outside, I simply close the windows, and then I am no longer disturbed.<br>I-18. Sometimes, I really feel at the mercy of the noise.<br>I-19. If it is very loud, I just mentally switch off.<br>I-20. I do not hear the noise anymore.<br>I-21. I have accepted the fact that the noise is here.

**<Source-specific> noise-related disturbances**	**Affective evaluation, attitudes**	**Perception of loss of control, lack of coping capacity**
	outdoor stay in the garden, on the terrace or on the balcony.<br>Not included in the EFA for aircraft noise annoyance but part of the assessed statements of expectations (Cronbach's of the 4-items expectation scale: 〈 = 0.74)<br>● The airport improves the regional development<br>● The air traffic brings new jobs to the region<br><br>*Attributes of <noise source>*<br>What do you think about <noise source>? Do you think the <noise source> is … (1) not, (2) a little bit, (3) moderately, (4) rather, (5) very …<br>I-12. useful<br>I-13. dangerous for me<br>I-14. comfortable for users<br>I-15. environmental harmful	

Note. <source-specific> refers either to aircraft, road traffic, or railway noise, depending on the focus of the NORAH sub-studies (air: FRA-air, BER-air, CGN-air, STR-air; road: FRA-road; rail: FRA-rail).

Table S2: Items of the non-acoustical variables correlated with noise annoyance (in addition to the list of items in Table S1).

**Variables**	**Items**	**Cronbach's Alpha (in samples at Frankfurt Airport)**
Trust in authorities - air	Do you think that … do everything they can to reduce noise annoyance due to aircraft in the population?<br><br>(1) aircraft manufactures<br>(2) airlines<br>(3) the airport operator (Fraport AG),<br>(4) municipalities<br>(5) the Federal State Government of Hess,<br>*not included in the score 'trust in authorities':*<br>(6) the regional Aircraft Noise Commission<br>(7) German Air Traffic Control<br>(8) the regional dialogue forum 'Forum Airport & Region'<br>(9) the Aircraft Noise Commissionar<br>(10) the Federal Aviation Office<br><br>Response scale: <authority> endeavours (1) not; (2) a little; (3) moderately; (4) fairly; (5) very	0.84

**Variables**	**Items**	**Cronbach's Alpha (in samples at Frankfurt Airport)**
Trust in authorities - road	Do you think that … do everything they can to reduce noise annoyance due to road traffic in the population?<br><br>(1) car manufacturers<br>(2) regional public transport companies<br>(3) municipalities<br>(4) the Federal State Government<br>(5) car drivers<br><br>Response scale: <authority> endeavours (1) not; (2) a little; (3) moderately; (4) fairly; (5) very	0.81
Trust in authorities – railway	Do you think that … do everything they can to reduce noise annoyance due to rail traffic in the population?<br><br>(1) Deutsche Bahn AG (German railway)<br>(2) municipalities<br>(3) the Federal State Government<br><br>Response scale: <authority> endeavours (1) not; (2) a little; (3) moderately; (4) fairly; (5) very	0.80
Perceived procedural fairness – air	(1) I think that aircraft noise is distributed fairly amongst all residents;<br>(2) When decisions concerning aircraft noise are being made, I have opportunities to express my views to the relevant people;<br>(3) I have the chance to appeal decisions that I consider to be wrong;<br>(4) Decisions concerning aircraft noise are explained and justified to me in detail.<br><br>Response scale: Agree (1) not; (2) a little; (3) moderately; (4) fairly; (5) very	0.67

**Variables**	**Items**	**Cronbach's Alpha (in samples at Frankfurt Airport)**
SF8 Physical Component Summary	(1) Overall, how would you rate your health in the past 4 weeks?<br>(2) During the past 4 weeks, how much did physical health problems limit your usual physical activities (such as walking or climbing stairs)?<br>(3) During the past 4 weeks, how much difficulty did you have doing your daily work, both at home and away from home, because of your physical health?<br>(4) How much bodily pain have you had in the past 4 weeks?<br>(5) During the past 4 weeks, how much energy did you have?	
SF8 Mental Component Summary	(6) During the past 4 weeks, how much did your physical health or emotional problems limit your usual social activities with family or friends?<br>(7) During the past 4 weeks, how much have you been bothered by emotional problems (such as feeling anxious, depressed or irritable)?<br>(8) During the past 4 weeks, how much did personal or emotional problems keep you from doing your usual work, school or other daily activities?	
Noise sensitivity (single item from NoiSeq)	I-7. I am sound-sensitive.<br><br>Response scale:<br>(1) strongly agree; (2) slightly agree; (3) slightly disagree; (4) strongly disagree.	

2. Confirmatory factory analyses (CFA)

2.1 CFA for aircraft noise annoyance at Frankfurt Airport (FRA-air)

Table S3. Items MIAS scale (FRA-air, *N* = 3508)

	Dist1	Disturb talk/phone
F1	Dist2	Disturb radio TV
	Dist3	Disturb concentration
Annoyance	Annoy	Annoyance (aircraft)
	Cope 1	Protect against noise (recoded)
F2	Cope 2	Close windows (recoded)
	Cope 3	At the mercy of the noise

Table S4. Additional information - Final Factor loadings CFA (FRA-air, *N* = 3508)

**Item**	**Model A**	**Model C**	
		**F1**	**F2**
Dist1	0.918	0.888	
Dist2	0.898	0.863	
Dist3	0.904	0.933	
Annoy	0.801		
Cope1	0.529		0.637
Cope2	0.565		0.640
Cope3	0.698		0.863

Note. Standardized factor loads, wave 3 (*N* = 3508, with imputed data, 0); * $p < 0.001$. Model A = MIAS, 1 factor; model C = CFA, three indicators and residual co-variances

Table S5. Fit indices of CFA (FRA-air, *N* = 3508)

**Model**	χ^2	*df*	*p*	*CFI*	*RMSEA (90% CI)*	*SRMR*	*AIC*
Model A	1582.786	14	< 0.001	0.878	0.179 (0.171-0.186)	0.074	68948.858
Model B	100.413	11	< 0.001	0.993	0.048 (0.040-0.057)	0.023	67086.043
Model C	70.992	10	< 0.001	0.995	0.042 (0.033-0.051)	0.012	67057.113

Note. χ^2: Chi-square-test, *df*: degrees of freedom, *p* = probability of error, *CFI*: comparative fit index, *RMSEA*: root mean square error of approximation, 90% *CI* = 90% Confidence interval, *SRMR*: standardized root mean square residual values, *AIC*: Akaike information criterion.

Table S6. Factors´ psychometric adequacy (MIAS scale FRA-air, *N* = 3508)

**Construct**	*CR*	*α*	*AVE*	**1**	**2**	**3**
Disturbance	0.92	0.94	0.80	---		
Lack of coping capacity	0.76	0.79	0.52	0.79***	---	
Annoyance (single item)	---	---	---	0.80***	0.77***	---

Note. *AVE* = average variance extracted; *CR* = composite reliabilities, α = Cronbach's Alpha, the remaining values indicate correlations between factors. *** $p < 0.001$.

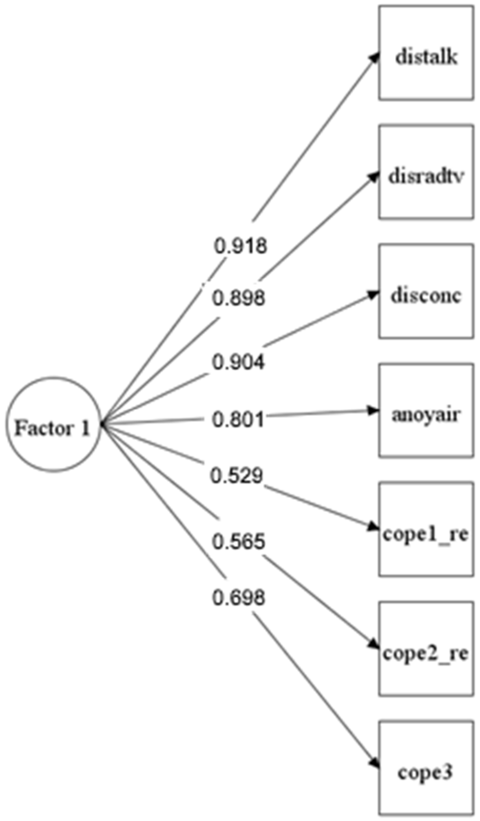

Figure S1. CFA model A: Aircraft (FRA-air; *N* = 3508)

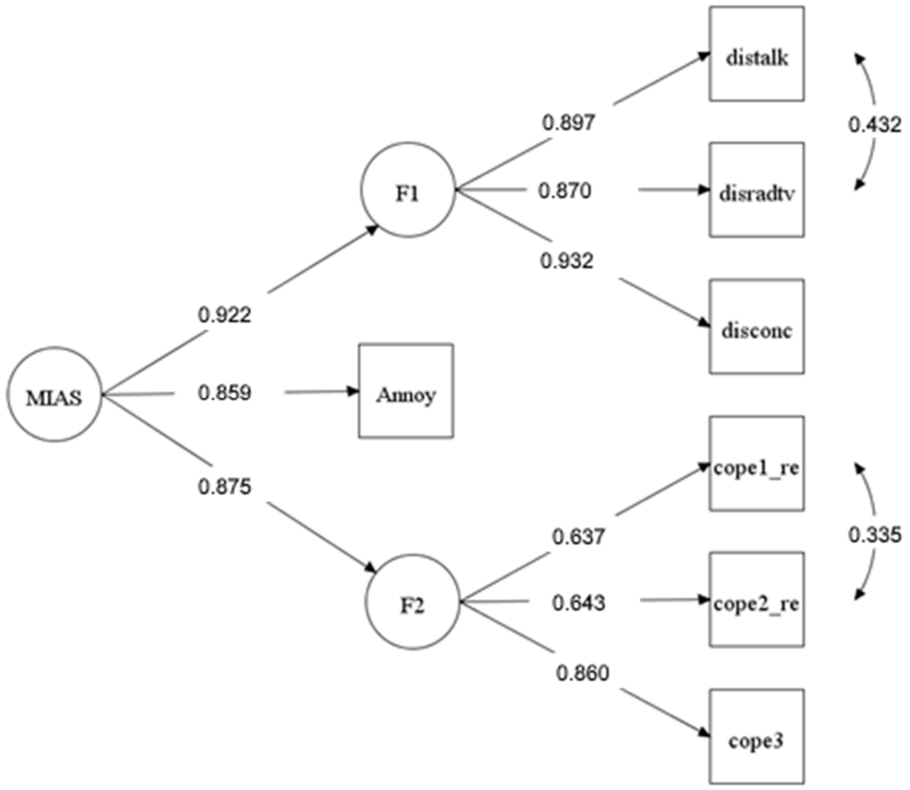

Figure S2. CFA-model B: Aircraft (FRA-air; *N* = 3508)

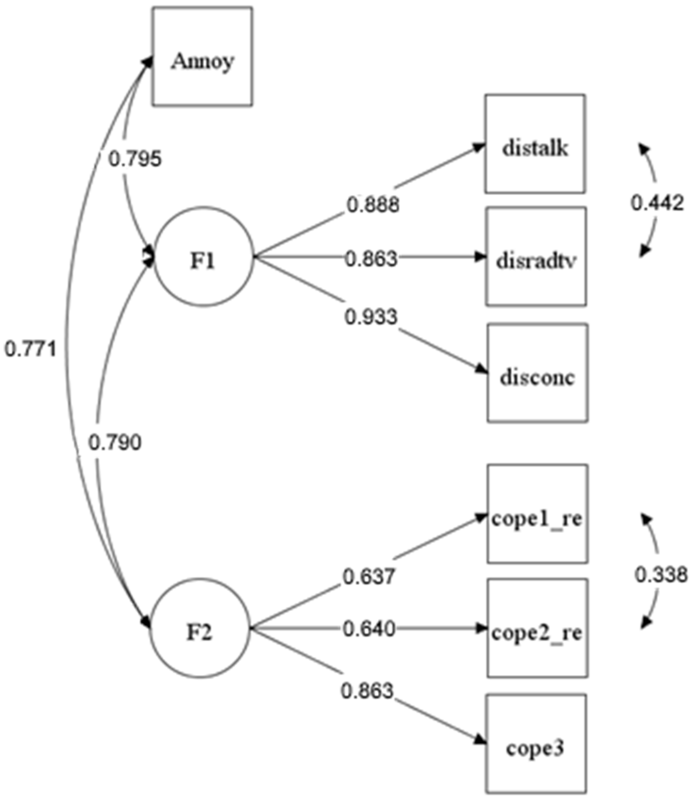

Figure S3. CFA-model C: Aircraft (FRA-air; *N* = 3508)

2.2 CFA for aircraft noise annoyance at different airports

Table S7. Comparison of fit indices of CFA for the multiple-item aircraft noise annoyance scale (MIAS-air) conducted with data of the samples FRA, BER, CGN, and STR.

	Model	χ^2	*df*	*p*	*CFI*	*RMSEA* (90% CI)	*SRMR*	*AIC*
'FRA-air'	A	1582.786	14	< 0.001	0.878	0.179 (0.171-0.186)	0.074	68948.858
(*n* = 3508)	B	100.413	11	< 0.001	0.993	0.048 (0.040-0.057)	0.023	67086.043
'BER-air'	A	2037.111	14	< 0.001	0.863	0.161 (0.156-0.167)	0.087	106269.291
(*n* = 5548)	B	245.345	11	< 0.001	0.984	0.062 (0.055-0.069)	0.038	104097.298
'CGN-air'	A	1360.386	14	< 0.001	0.857	0.180(0.172-0.189)	0.089	59403.792
(*n* = 2954)	B	103.090	11	< 0.001	0.990	0.053 (0.044-0.063)	0.034	57867.201
'STR-air'	A	693.917	14	< 0.001	0.865	0.157 (0.147-0.167)	0.090	37849.159
(*n* = 1979)	B	97.945	11	< 0.001	0.983	0.063 (0.052-0.075)	0.033	37092.805

Note. χ^2: Chi square test, *df*: degrees of freedom, *p* = probability of error, *CFI*: comparative fit index, *RMSEA*: root mean square error of approximation, 90% *CI* = 90% confidence interval, *SRMR*: standardized root mean square residual values, *AIC*: Akaike information criterion.

2.3 CFA for railway noise annoyance (FRA-rail)

Table S8. Items MIAS scale (railway) - FRA-rail, $N = 3307$

	Dist1	Disturb talk/phone
F1	Dist2	Disturb radio TV
	Dist3	Disturb concentration
Annoyance	Annoy	Annoyance (railway)
	Cope 1	Protect against noise (recoded)
F2	Cope 2	Close windows (recoded)
	Cope 3	At the mercy of the noise

Table S9. Additional information - Final Factor loadings CFA (FRA-rail, $N = 3307$)

**Item**	**Model A**	**Model C**	
		**F1**	**F2**
Dist1	0.924	0.902	
Dist2	0.921	0.901	
Dist3	0.908	0.928	
Annoy	0.731		
Cope1	0.298		0.551
Cope2	0.279		0.518
Cope3	0.464		0.926

Note. Standardized factor loads, wave 2 ($N = 3307$, with imputed data, *FIML*); * $p < 0.001$. Model A = MIAS, 1 factor; model C = CFA, three indicators and residual co-variances

Table S10. Fit indices of CFA (Railway, FRA-rail, $N = 3307$)

Model	χ^2	df	p	CFI	RMSEA (90% CI)	SRMR	AIC
Model A	1639.183	14	< 0.001	0.797	0.187 (0.180-0.195)	0.124	62445.933
Model B	152.457	11	< 0.001	0.982	0.062 (0.054-0.071)	0.033	60364.530
Model C	36.812	10	< 0.001	0.997	0.028 (0.019-0.039)	0.008	60231.457

Note. χ^2: Chi-square-test, *df*: degrees of freedom, p = probability of error, *CFI*: comparative fit index, *RMSEA*: root mean square error of approximation, 90% *CI* = 90% Confidence interval, *SRMR*: standardized root mean square residual values, *AIC*: Akaike information criterion.

Table S11. Factors´ psychometric adequacy (MIAS scale railway, FRA-rail, $N = 3307$)

**Construct**	***CR***	***α***	***AVE***	**1**	**2**	**3**
Disturbance	0.94	0.94	0.83	---		
Lack of coping capacity	0.72	0.76	0.48	0.48***	---	
Annoyance (single item)	---	---	---	0.73***	0.51***	---

Note. AVE = average variance extracted; *CR* = composite reliabilities, α = Cronbach's Alpha, the remaining values indicate correlations between factors. *** $p < 0.001$.

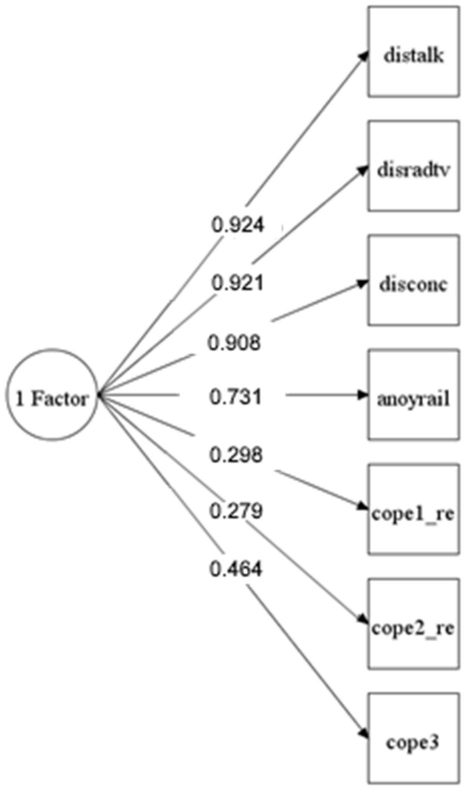

Figure S4. CFA-model A: Railway (FRA-rail, *N* = 3307)

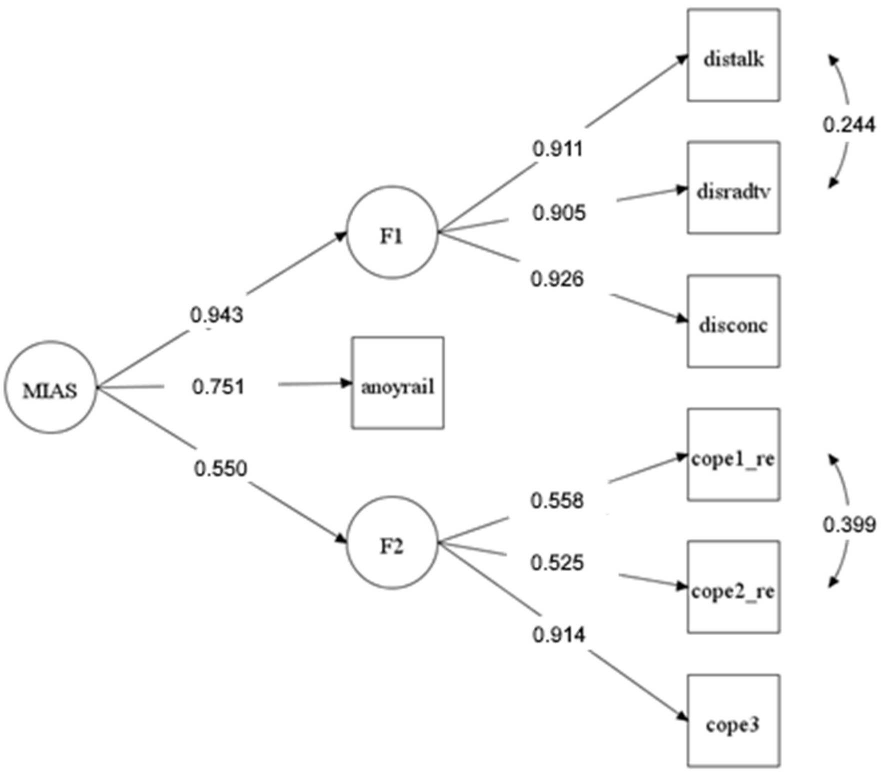

Figure S5. CFA-model B: Railway (FRA-rail, *N* = 3307)

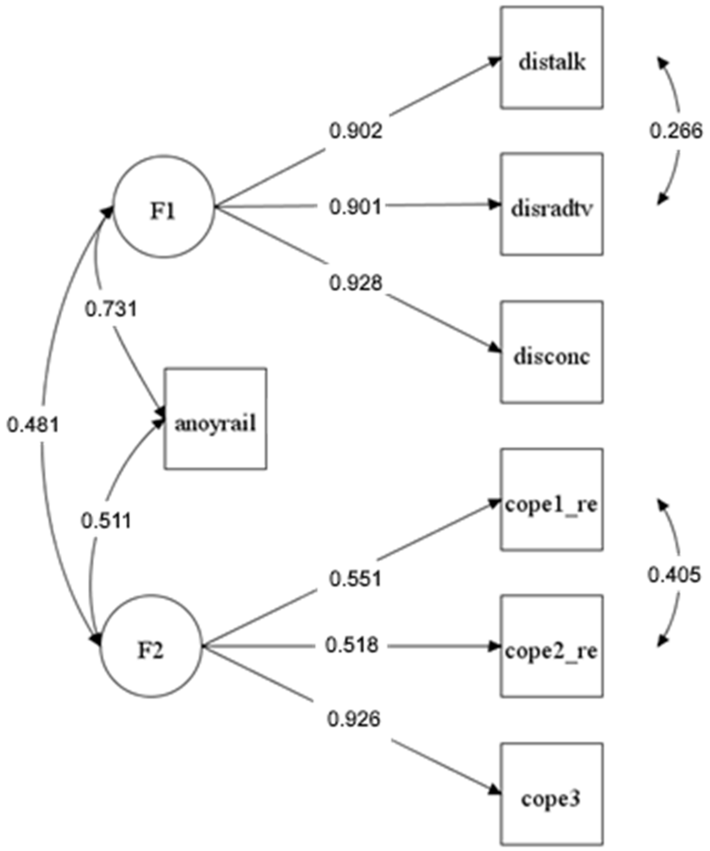

Figure S6. CFA-model C: Railway (FRA-rail, *N* = 3307)

2.4 CFA for road noise (FRA-road)

Table S12. Items MIAS scale (road, FRA-road, *N* = 3172)

	Dist1	Disturb talk/phone
F1	Dist2	Disturb radio TV
	Dist3	Disturb concentration
Annoyance	Annoy	Annoyance (railway)
	Cope 1	Protect against noise (recoded)
F2	Cope 2	Close windows (recoded)
	Cope 3	At the mercy of the noise

Table S13. **Additional information -** Final Factor loadings CFA (FRA-road, *N* = 3172)

**Item**	**Model A**	**Model C**	
		**F1**	**F2**
Dist1	0.897	0.853	
Dist2	0.889	0.849	
Dist3	0.875	0.912	
Annoy	0.694		
Cope1	0.290		0.473
Cope2	0.261		0.445
Cope3	0.500		0.961

Note. Standardized factor loads, wave 2 (*N* = 3172, with imputed data, *FIML*); * *p* < 0.001. Model A = MIAS, 1 factor; model C = CFA, three indicators and residual co-variances

Table S14. Fit indices of CFA (road, FRA-road, $N = 3172$)

Model	χ^2	*df*	*p*	*CFI*	*RMSEA (90% CI)*	*SRMR*	*AIC*
Model A	1569.665	14	< 0.001	0.791	0.187 (0.179-0.195)	0.114	58908.756
Model B	273.560	11	< 0.001	0.965	0.087 (0.078-0.096)	0.046	57208.810
Model C	64.728	10	< 0.001	0.993	0.042 (0.032-0.051)	0.013	56983.212

Note. χ^2: Chi-square-test, *df*: degrees of freedom, *p* = probability of error, *CFI*: comparative fit index, *RMSEA*: root mean square error of approximation, 90% *CI* = 90% Confidence interval, *SRMR*: standardized root mean square residual values, *AIC*: Akaike information criterion.

Table S15. Factors´ psychometric adequacy (MIAS scale model road, FRA-road, $N = 3172$)

Construct	*CR*	*α*	*AVE*	1	2	3
Disturbance	0.91	0.92	0.76	---		
Lack of coping capacity	0.68	0.73	0.45	0.50***	---	
Annoyance (single item)	---	---	---	0.70***	0.51***	---

Note. *AVE* = average variance extracted; *CR* = composite reliabilities, *α* = Cronbach's Alpha, the remaining values indicate correlations between factors. *** $p < 0.001$.

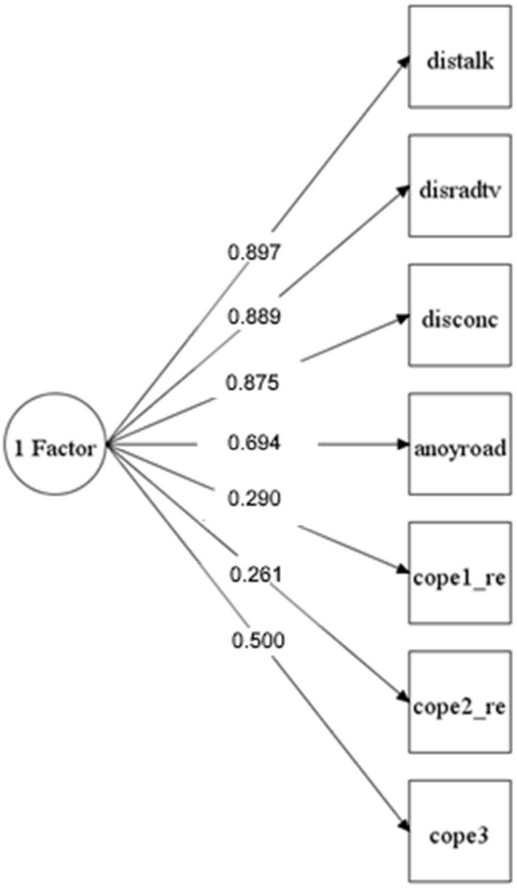

Figure S7. CFA-model A: Road (FRA-road, $N = 3172$)

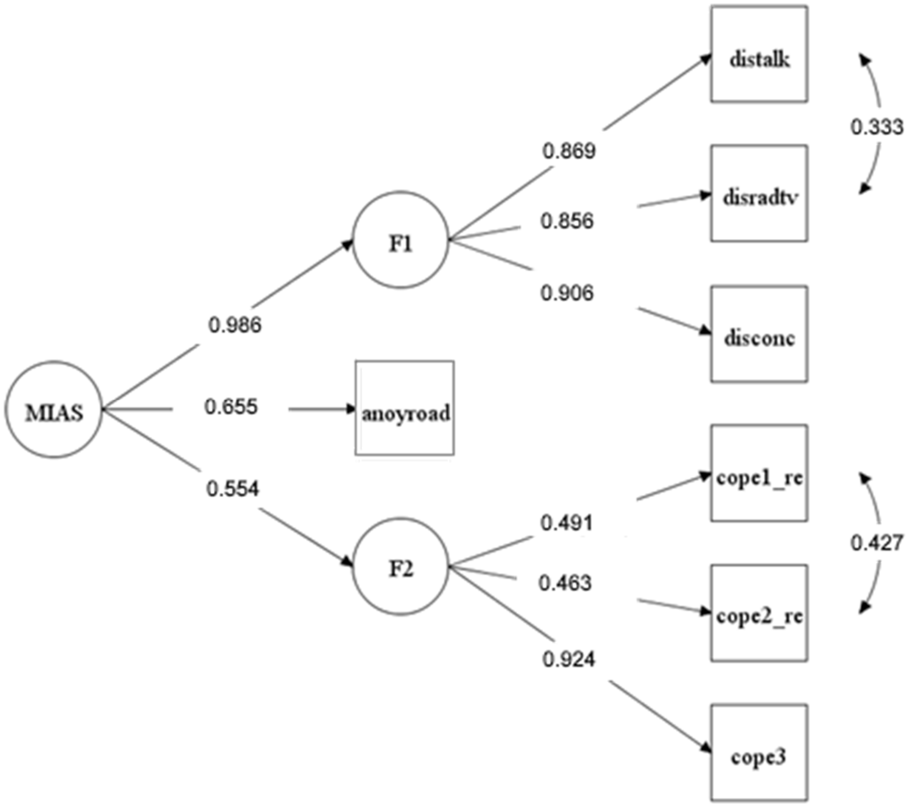

Figure S8. CFA-model B: Road (FRA-road, *N* = 3172)

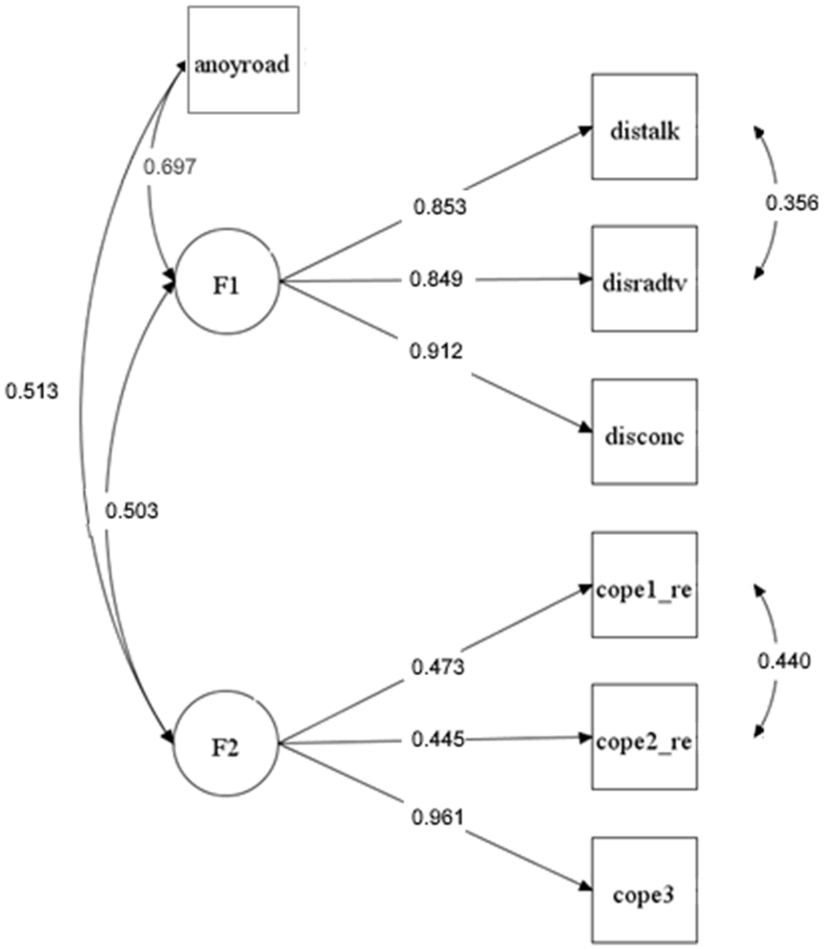

Figure S9. CFA-model C: Road (FRA-road, *N* = 3172)

© 2018 by the authors. Submitted for possible open access publication under the terms and conditions of the Creative Commons Attribution (CC BY) license (http://creativecommons.org/licenses/by/4.0/).
